# Rab18 Binds to Hepatitis C Virus NS5A and Promotes Interaction between Sites of Viral Replication and Lipid Droplets

**DOI:** 10.1371/journal.ppat.1003513

**Published:** 2013-08-01

**Authors:** Shadi Salloum, Hongliang Wang, Charles Ferguson, Robert G. Parton, Andrew W. Tai

**Affiliations:** 1 Division of Gastroenterology, Department of Internal Medicine, University of Michigan, Ann Arbor, Michigan, United States of America; 2 The University of Queensland, Institute for Molecular Bioscience and Centre for Microscopy and Microanalysis, Brisbane, Queensland, Australia; 3 Division of Gastroenterology, Department of Internal Medicine, Ann Arbor Veterans Administration Health System, Ann Arbor, Michigan, United States of America; University of Alabama at Birmingham, United States of America

## Abstract

Hepatitis C virus (HCV) is a single-stranded RNA virus that replicates on endoplasmic reticulum-derived membranes. HCV particle assembly is dependent on the association of core protein with cellular lipid droplets (LDs). However, it remains uncertain whether HCV assembly occurs at the LD membrane itself or at closely associated ER membranes. Furthermore, it is not known how the HCV replication complex and progeny genomes physically associate with the presumed sites of virion assembly at or near LDs. Using an unbiased proteomic strategy, we have found that Rab18 interacts with the HCV nonstructural protein NS5A. Rab18 associates with LDs and is believed to promote physical interaction between LDs and ER membranes. Active (GTP-bound) forms of Rab18 bind more strongly to NS5A than a constitutively GDP-bound mutant. NS5A colocalizes with Rab18-positive LDs in HCV-infected cells, and Rab18 appears to promote the physical association of NS5A and other replicase components with LDs. Modulation of Rab18 affects genome replication and possibly also the production of infectious virions. Our results support a model in which specific interactions between viral and cellular proteins may promote the physical interaction between membranous HCV replication foci and lipid droplets.

## Author Summary

Hepatitis C virus (HCV) chronically infects about 170 million people worldwide and can ultimately lead to liver failure and liver cancer. HCV, like other RNA viruses, exploits cellular proteins and membranes to promote their own replication and virion production. In particular, HCV replication occurs at membranes derived from the endoplasmic reticulum, while HCV virion assembly is believed to occur at or near cellular lipid droplets. In this work, we report that Rab18, a lipid droplet-associated cellular protein, binds to the viral protein NS5A, and that the silencing of Rab18 reduces the association of other HCV replication complex components with lipid droplets. These data are consistent with a model in which Rab18 promotes the physical interaction between sites of viral replication to lipid droplets. We also speculate that Rab18 may help to link sites of viral replication to sites of virion assembly. Understanding how viruses exploit cellular proteins may result in new methods of disrupting viral infection.

## Introduction

Hepatitis C virus (HCV) is a positive-sense RNA virus in the family Flaviviridae that is estimated to chronically infect up to 170 million people worldwide. The 9.6 kb genome encodes three structural and seven nonstructural proteins. One of these nonstructural proteins, NS5A, is an RNA-binding phosphoprotein essential for both viral replication and viral particle assembly [1]. It is composed of a N-terminal amphipathic helix that mediates membrane association [2]–[4] followed by three domains separated by two low-complexity sequences [5]. Domain I is responsible for NS5A dimerization [6] and has been proposed to contribute to RNA binding [7], [8]. A role for this domain in HCV RNA replication has been supported by the finding that many adaptive mutations that enhance HCV replication in cell culture map to Domain I [9], [10], In contrast, the majority of Domain II and the entirely of Domain III are dispensable for RNA replication, while deletion of Domain III virtually abolishes viral particle assembly [1].

How NS5A supports both viral RNA replication and particle assembly remains incompletely understood. NS5A has been reported to interact with the core [11], NS2 [12], [13], and NS5B [14], [15] viral proteins. Furthermore, it has been reported to interact with many host proteins, such as PI4KA [16]–[18], VAP-A [19], VAP-B [20], and FKBP8 [21]. As NS5A is believed to lack intrinsic enzymatic activity, the main function of NS5A may be to coordinate interactions among viral and host proteins.

Indirect evidence suggests that NS5A function might be regulated by its intracellular localization. Only a small fraction of HCV nonstructural proteins appears to be associated with HCV replication complexes in protease-resistant membranes [22], [23], raising the possibility that some of the HCV nonstructural proteins have additional functions in the cell outside of the HCV replication complex. In particular, HCV NS5A has been reported to localize to ER and lipid droplet (LD) membranes [2], [24]–[26]. Interestingly, a small-molecule NS5A inhibitor induces NS5A redistribution from ER to LDs [27], suggesting that the intracellular trafficking of NS5A might be regulated.

Lipid droplets are lipid storage organelles composed of a core of neutral lipids, sterols, and sterol esters surrounded by a phospholipid monolayer. In addition to NS5A, HCV core protein localizes to LDs [28]. The core-LD association is thought to be essential for virion assembly [26], [29], [30]; however, it remains uncertain whether virion assembly actually occurs on the LD membrane itself or on membranes closely associated with LDs. In addition, it is not known how viral RNA genomes synthesized at replicase complexes are transferred to sites of particle assembly. Recent work has demonstrated that LDs, rather than being simple lipid storage depots, are dynamic, motile organelles that interact with other intracellular membranes such as the ER and possibly also mitochondria [31]. LD functions and interactions with intracellular membranes may be regulated by proteins that specifically associate with the LD surface. For example, the Rab18 small GTPase localizes to LDs, and Rab18-LD association is enhanced by agents that stimulate lipolysis [32]. Furthermore, Rab18 overexpression has been shown to promote the association of LDs with ER membranes [33].

Using a proteomic strategy to identify novel host proteins that interact with HCV NS5A in HCV-infected cells, we have found that NS5A associates with Rab18. Furthermore, Rab18 modulation affects NS5A localization to LDs. Rab18 also appears to regulate the association of NS5A-positive membranes and other HCV replicase components with lipid droplets, suggesting a model in which binding of Rab18 to NS5A physically recruits sites of HCV replication to LDs.

## Results

### Identification of novel NS5A-associated host proteins in HCV-infected cells

**Figure 1:**
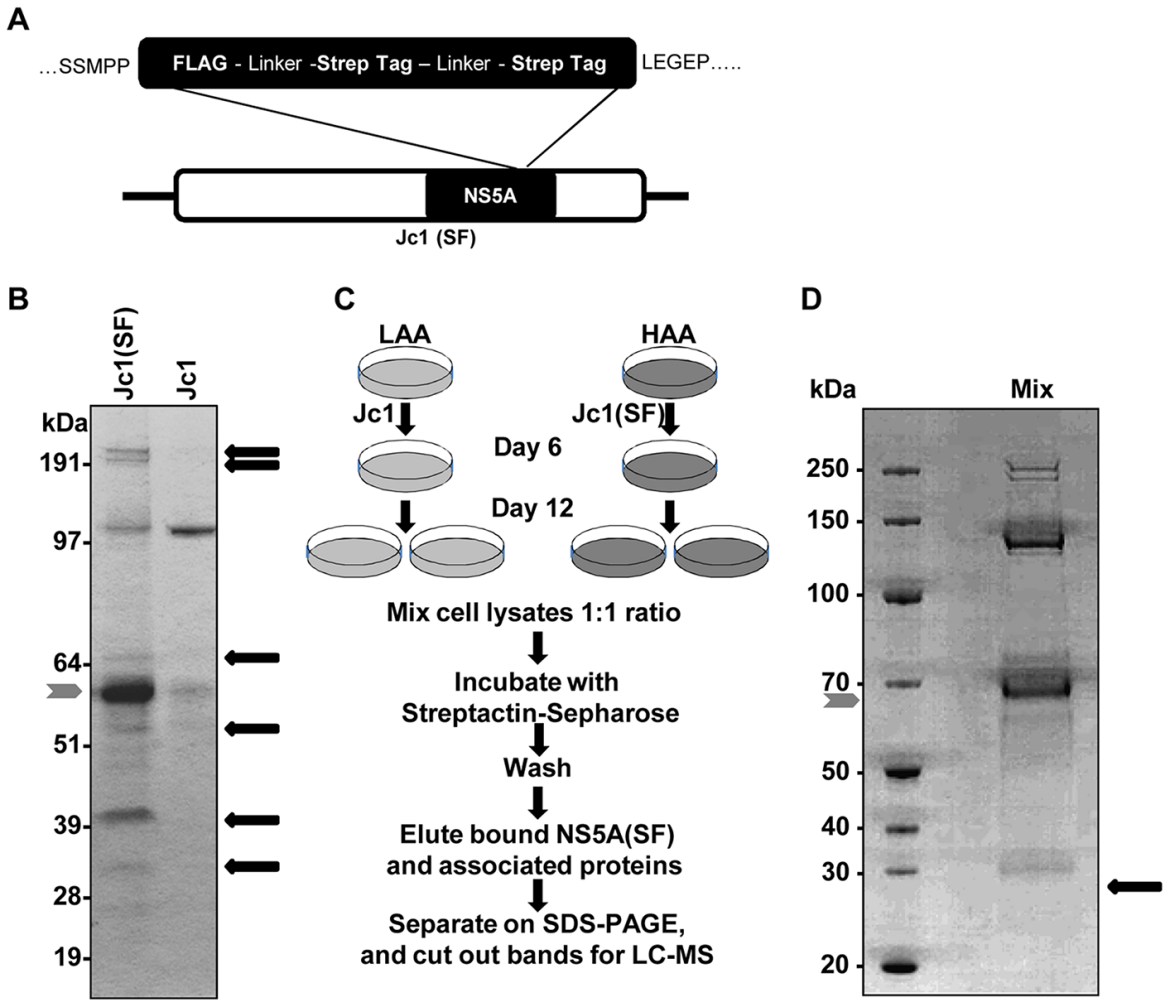
Identification of NS5A-binding host proteins using a proteomic strategy. **A.** Diagram of Jc1(SF) showing location of FLAG and tandem Strep-Tags in domain III of NS5A. **B.** Detection of proteins specifically associated with NS5A(SF). Huh7.5.1 cells were infected with Jc1(SF) (left lane) and untagged wild-type Jc1 (right lane). Cell lysates were incubated with Streptactin-Sepharose and the affinity matrix was washed extensively followed by elution with biotin. Eluted proteins were separated by SDS-PAGE and visualized by colloidal Coomassie Blue staining. The position of NS5A(SF) is indicated by the gray arrowhead. Proteins that specifically associate with NS5A(SF) are indicated by black arrows. **C.** Schematic showing strategy of affinity purification combined with Stable Isotope Labeling with Amino Acids in Cell Culture (SILAC). Huh 7.5.1 cells were metabolically labeled with medium containing normal “light” arginine and lysine amino acids (LAA) or “heavy” L-arg-^13^C_6_, ^15^N_4_ and L-lys-^13^C_6_, ^15^N_2_ (HAA) for 6 days before infecting LAA-labeled cells with Jc1 and HAA-labeled cells with Jc1(SF) virus. Cells were harvested at day 6 post infection. Equal amounts of protein from HAA and LAA-labeled lysates were mixed and then subjected to Streptactin affinity purification as described above. **D.** Lysates from LAA-labeled, Jc1-infected cells and HAA-labeled, Jc1(SF)-infected cells were mixed and subjected to Streptactin affinity purification. The entire eluate was concentrated by precipitation, separated by SDS-PAGE and stained with Coomassie Blue prior to gel slice excision for mass spectrometry. The expected position of Rab18 is indicated with an arrow, although no discrete band is visible on this gel by Coomassie Blue staining.

NS5A, which lacks known intrinsic enzymatic activity, is thought to exert its functions through interactions with host and viral proteins. We therefore sought to identify novel host protein-NS5A interactions, which in turn should yield new insights into the HCV life cycle. As depicted in Figure 1A and described in previously published work [17], we inserted a small tandem affinity purification tag consisting of a tandem Strep-tag II and a FLAG tag into domain III of NS5A at a site previously shown to be tolerant of heterologous insertions such as GFP [34]. The chimeric genotype 2a Jc1 (J6/JFH1) genome, which is fully infectious in cell culture, was used as the backbone [35], [36]. This tag was genetically stable and following cell culture adaptation, titers of the tagged Jc1(SF) virus were comparable to those of wild-type Jc1 [17].

We initially attempted to perform tandem affinity purification of tagged NS5A(SF) from Jc1(SF)-infected Huh7.5.1 hepatoma cells using sequential anti-FLAG and Streptactin-Sepharose affinity purification steps [37]. However, the yield of NS5A(SF) following anti-FLAG affinity purification was insufficient for downstream mass spectrometry analysis (data not shown), and therefore we used single-step Streptactin-Sepharose affinity purification of NS5A(SF). In pilot experiments, we found that Streptactin-Sepharose failed to bind to NS5A(SF) in cellular homogenates of Jc1(SF)-infected cells prepared in the absence of detergent, but did bind to NS5A(SF) in detergent lysates (data not shown).

Figure 1B demonstrates that a number of proteins specifically copurified with NS5A(SF) from Jc1(SF)-infected cells (left lane), but not from wild-type Jc1-infected cells (right lane). On the other hand, we did observe nonspecific interactions with Streptactin-Sepharose from untagged Jc1-infected cells, which was not unexpected from single-step affinity purification. To minimize the false-positive identification of NS5A interacting proteins in subsequent mass spectrometry, we added Stable Isotope Labeling with Amino Acids in Cell Culture (SILAC; Figure 1C) [38]. Huh7.5.1 cells were either cultured in medium with normal “light” amino acids (LAA) or with medium containing only “heavy” ^13^C_6_, ^15^N_4_ L-arginine and ^13^C_6_, ^15^N_2_ L-lysine (HAA), resulting in 97% labeling of the cellular proteome with heavy L-arg and L-lys in pilot studies (data not shown). Cells labeled with LAA were infected with wild-type Jc1, while cells labeled with HAA were infected with Jc1(SF). Equal amounts of protein in lysates from Jc1-infected and Jc1(SF)-infected cells were mixed together and then subjected to affinity purification of NS5A(SF) and bound proteins using Streptactin-Sepharose. Eluted proteins were separated by SDS-PAGE and stained with Coomassie Blue (Figure 1D); gel slices were subjected to trypsin digestion and tryptic peptides identified by LC-MS/MS. Peptides containing “heavy” L-lys and/or L-arg from Jc1(SF)-infected cells have a higher mass and can be resolved by mass spectrometry from those from Jc1-infected cells so that their respective intensities can be quantitated. Therefore, the ratios of heavy∶light peptide intensities permits discrimination among proteins specifically bound to NS5A(SF) (heavy∶light peptide ratio >1), proteins nonspecifically bound to Streptactin-Sepharose (heavy∶light peptide ratio ∼1), and environmental contaminants introduced during protein purification and gel preparation (e.g. keratins from skin; heavy∶light peptide ratio <<1).

As shown in Table 1, this strategy successfully identified multiple known host proteins known to interact with NS5A or with the HCV life cycle, such as PI4KA, cyclophilin A, casein kinase 1, VAPA/h-VAP33, FKBP8, and B-IND1/PTPLAD1, thus validating this strategy for the discovery of novel NS5A-interacting cellular proteins. We also identified Rab18 as a previously unknown NS5A-binding protein with a heavy∶light peptide ratio of 8.16, which was higher than those of FKBP8 (4.83) and B-IND1/PTPLAD1 (3.24) and similar to that of cyclophilin A. No peptides derived from viral proteins were identified by mass spectrometry.

**Table 1:** Host proteins already known to interact with NS5A and/or the HCV life cycle identified by SILAC and GeLC-MS.

Host Factor	H/L peptide ratio	Function	PEP	Heavy peptide intensity	Light peptide intensity
PI4KA	275	Catalyzes the production of PI 4-phosphate from PI (e.g. [16], [41], [45])	0	6.3×10^8^	2.3×10^6^
COPA	29.11	Subunit of the COPI coatomer complex [41]	2.2×10^−16^	4.0×10^5^	1.4×10^4^
CSNK1A1 (casein kinase I)	15.3	Phosphorylates NS5A [65]	5×10^−19^	2.1×10^5^	1.4×10^4^
ELAVL1	11.7	RNA binding protein [66]	1.7×10^−141^	5.2×10^7^	4.49×10^6^
VAP-A	11.0	Required for HCV replication, involved in cellular membrane trafficking [67]	7.5×10^−33^	1×10^7^	9.5×10^5^
CYPA (cyclophilin A)	8.75	Peptidyl-prolyl isomerase [68], [69]	9×10^−21^	4×10^6^	4.6×10^5^
Rab18	8.16	Lipid droplet-associated Rab protein [32], [70]	6.1×10^−19^	6.1×10^5^	7.4×10^4^
FKBP8	4.83	Immunophilin family; proposed to function as cochaperone of Hsp90 [21]	2.2×10^−13^	2.4×10^5^	5.1×10^4^
DHCR24	3.42	Cholesterol biosynthesis [71]	7.8×10^−3^	3.7×10^5^	1.1×10^5^
B-IND1/PTPLAD1	3.24	Interacts with NS5A and FKBP8 [72]	4.1×10^−25^	6.2×10^5^	1.9×10^5^

Proteins that specifically bind to NS5A(SF) will have incorporated heavy amino acids and thus will have a high heavy∶light (H/L) peptide ratio. PEP (Posterior error probability) refers to the probability that a peptide-spectrum match is incorrect. Intensity refers to total summed peptide intensity for the identified protein group.

### NS5A colocalizes with Rab18-positive lipid droplets in HCV-infected cells

**Figure 2:**
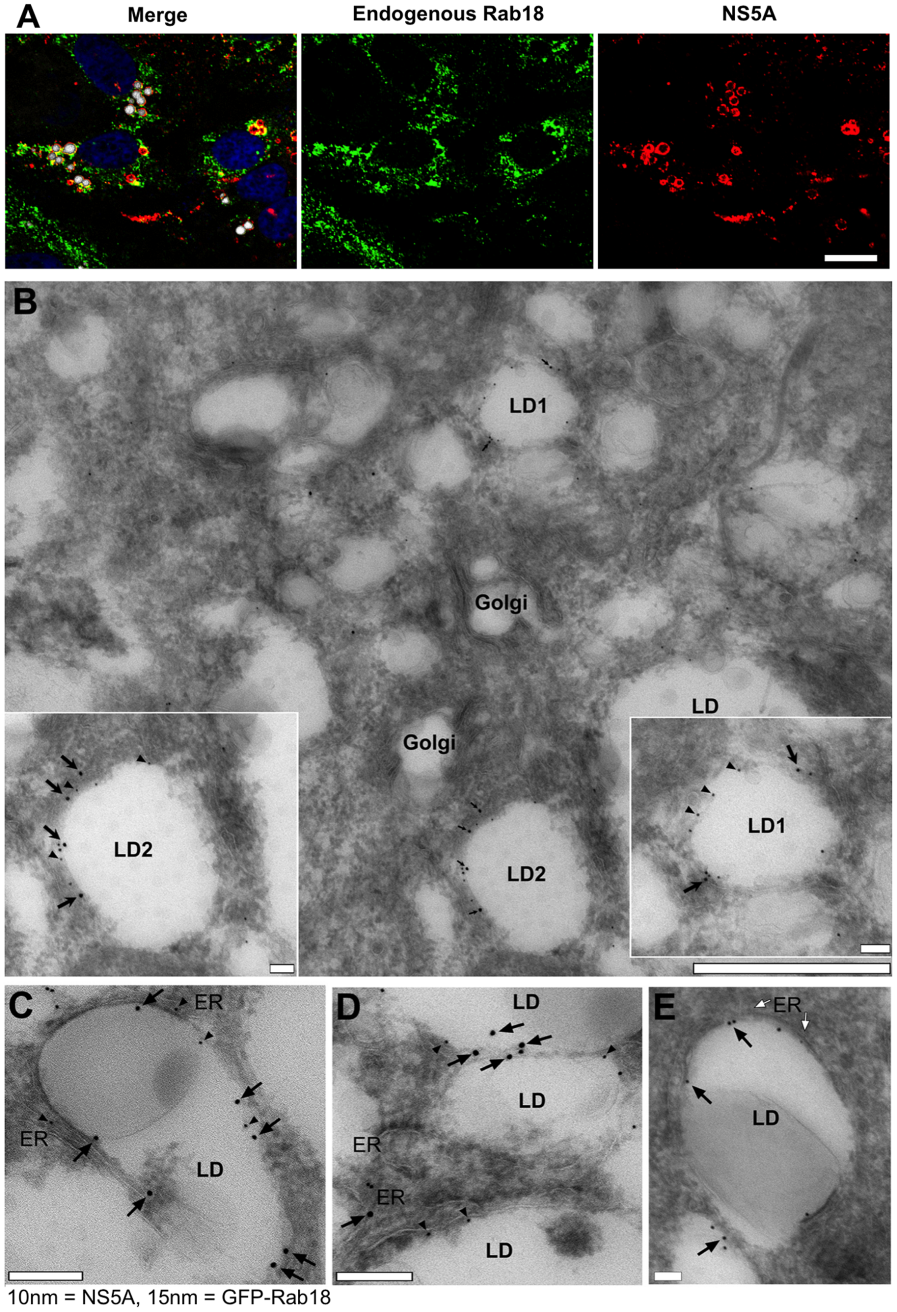
Colocalization of NS5A with Rab18-positive LDs in HCV-infected cells. **A.** Huh7.5.1 hepatoma cells were infected with JFH-1 and then immunolabeled for endogenous Rab18 (green) and NS5A (red). LDs were visualized by HCS LipidTOX Deep Red neutral lipid staining (false-colored white in these panels) and nuclei were counterstained with DAPI (blue). Bar, 10 µm. (**B–E**). Immunogold transmission electron microscopy of HCV-infected (**B–D**) or control uninfected (**E**) Huh7.5.1 cells stably expressing GFP-Rab18. Insets in panel B represent zoomed-in views of the lipid droplets LD1 and LD2. GFP-Rab18 is labeled with 15 nm gold particles (arrows), while NS5A is labeled with 10 nm gold particles (arrowheads). The white arrows in panel E point to putative ER membranes in close apposition to the LD surface. Bar in panel B, 1 µm; C–E and insets, 100 nm.

Rab18, a member of the Rab family of small GTPases, has not been previously shown to have a function in the HCV life cycle. Rab18 associates with lipid droplets [32], [33], which form a cellular compartment for the storage of neutral lipids and cholesteryl esters. Lipid droplets (LDs) are believed to be a site of HCV particle assembly [26], and NS5A interacts with LDs independently of core protein [24], [25], [27]. To further evaluate the significance of NS5A binding to Rab18, we proceeded to examine the localization of NS5A and endogenous Rab18 in HCV-infected cells. We first studied endogenous Rab18 to avoid possible artifacts due to Rab18 overexpression. Triple labeling of JFH-1 infected Huh7.5.1 cells with antibodies against NS5A, Rab18, and a fluorescent neutral lipid stain to visualize lipid droplets revealed NS5A staining around Rab18-positive LDs (Figure 2A). We determined that 59±19% of LDs stained positive for endogenous Rab18; of the Rab18^+^ LDs, 92±13% were also NS5A positive. Endogenous Rab18 and genotype 1 NS5A also displayed substantial colocalization in the OR6 cell line, which harbors a full-length genotype 1b HCV replicon with a *Renilla* luciferase reporter gene [39] (Figure S1A). The association of JFH-1 core protein with LDs has been described in detail; however, others have reported that Jc1 core protein is more closely associated with ER membranes at steady-state [30], [40]. We found that core protein appeared to localize around LDs in both JFH-1 and Jc1(SF)-infected cells (Figure S1B).

We next examined the distribution of NS5A with respect to Rab18 at the ultrastructural level in Huh7.5.1 cells stably expressing GFP-Rab18. Ultrathin frozen sections of control cells or cells infected with HCV for 5 days were double immunolabeled for GFP-Rab18 and for NS5A. Infected cells showed numerous electron-lucent Rab18-positive lipid droplets distributed throughout the cytoplasm (Figure 2B). NS5A and Rab18 showed colocalization on the surface of lipid droplets and on associated membranes, recognizable as rough ER in some areas (Figure 2C and D). In contrast, non-infected control cells showed GFP-Rab18 associated with ER and putative lipid droplets; labeling of GFP-Rab18 surrounding electron-lucent presumed LDs with closely apposed putative ER elements was a common observation in these cells (Figure 2E, white arrows). NS5A immunoreactivity was not observed in uninfected cells. A semiquantitative evaluation of immunogold labeling of LDs in infected cells showed that among a total of 105 analyzed LDs, 36 (34%) had Rab18 labeling. Of these 36 Rab18^+^ LDs, 34 (94%) were also labeled for NS5A. An additional 27 LDs (26%) were positive for NS5A and negative for Rab18 labeling. Quantitation of an independent labeling experiment gave similar results (30% of the LDs in infected cells had Rab18 labeling; of these Rab18^+^ LDs, 89% were also labeled for NS5A). These results suggest that the majority of Rab18^+^ lipid droplets are associated with HCV NS5A.

### Rab18 silencing reduces HCV replication

**Figure 3:**
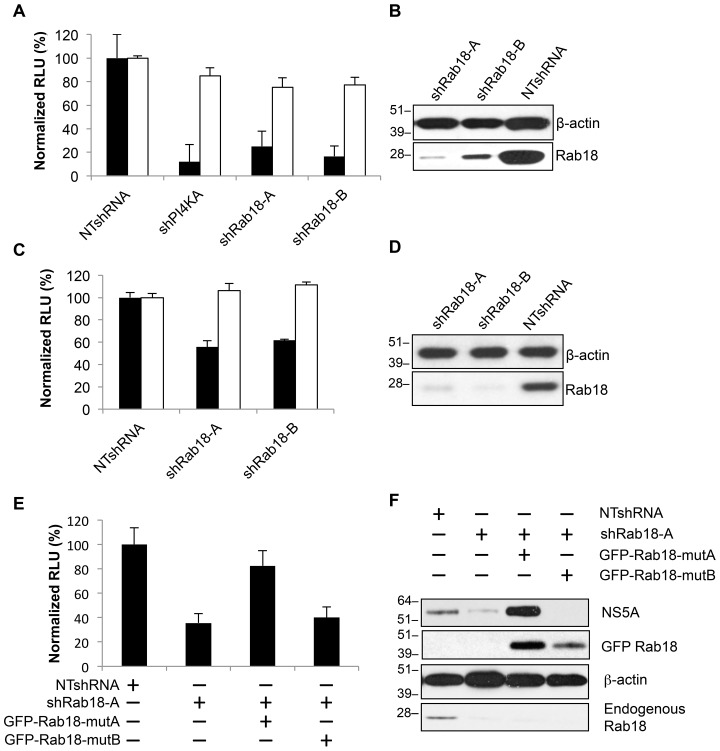
Rab18 silencing inhibits HCV replication. **A.** OR6 cells harboring a full-length genotype 1b replicon with a *Renilla* luciferase reporter were transduced with lentiviral shRNA vectors encoding a negative control nontargeting shRNA (NTshRNA), a positive control shRNA targeting PI4KA (shPI4KA), or two independent shRNAs targeting Rab18 (shRab18-A and -B). HCV replication was assessed by quantitation of *Renilla* luciferase activity (black bars) and cell viability was assessed by cellular ATP content (white bars). All values are normalized to OR6 cells transduced with NTshRNA and represent means ± SD of three independent experiments. **B.** Immunoblotting of the OR6 cells shown in panel A was performed for Rab18 and β-actin to confirm silencing of Rab18. **C.** Jc1/Gluc2A encoding a *Gaussia* luciferase reporter was used to infect Huh7.5.1 cells stably expressing NTshRNA, shRab18-A, or shRab18-B at an MOI of 1. 96 hr post-infection, *Gaussia* luciferase activity (black bars) and cellular ATP levels (white bars) were measured. All values are normalized to cells stably expressing NTshRNA and represent means ± SD of three independent experiments. **D.** Immunoblotting of the Huh7.5.1 cells shown in panel C was performed for Rab18 and β-actin to confirm silencing of Rab18. **E.** OR6 cells stably expressing GFP (first two bars), GFP-Rab18-mutA (resistant to silencing by shRab18-A; third bar), or GFP-Rab18-mutB (resistant to shRab18-B; fourth bar) were transduced with lentiviral shRNA vectors encoding a nontargeting shRNA (first bar) or shRab18-A (bars 2–4). HCV replication was assessed by quantitation of *Renilla* luciferase activity. All values are normalized to OR6 cells transduced with NTshRNA and represent means ± SD of three independent experiments. **F.** Immunoblotting of the OR6 cells shown in panel E was performed to confirm endogenous Rab18 knockdown and expression of GFP-Rab18 constructs.

Given the colocalization of NS5A with Rab18 at lipid droplets, we next proceeded to examine the effect of Rab18 silencing on the HCV life cycle. We first silenced Rab18 in the OR6 replicon cell line. OR6 replicon cells were transduced with lentiviral vectors encoding two independent shRNAs targeting Rab18 (denoted as shRab18-A and shRab18-B), a control nontargeting shRNA (NTshRNA), or a positive control shRNA targeting PI4KA (shPI4KA) [41]. As shown in Figure 3A, Rab18 silencing inhibited replication of the genotype 1b replicon compared to cells expressing a nontargeting shRNA (black bars). Cell viability, as monitored by determination of cellular ATP content, was not significantly affected by Rab18 silencing (Figure 3A, white bars). Immunoblotting demonstrated that Rab18 protein levels were markedly lower in cell lines expressing either Rab18 shRNA (Figure 3B).

We then studied the effect of Rab18 silencing on the replication of the fully infectious genotype 2a Jc1/Gluc2A virus bearing a secreted *Gaussia* luciferase reporter [36]. Jc1/Gluc2A replication was inhibited in Huh7.5.1 cell pools stably expressing Rab18-targeting shRNAs relative to cells expressing a nontargeting shRNA (Figure 3C, black bars). As observed with OR6 replicon cells, Rab18 silencing had no effect on cell viability (Figure 3C, white bars) and Rab18 protein knockdown was confirmed by immunoblotting (Figure 3D).

Although the observation that two independent Rab18 shRNAs both block HCV replication, we performed a rescue experiment to exclude the possibility that the inhibition of viral replication could be due to off-target effects. We generated two GFP-Rab18 mutants, one with silent mutations at the site targeted by shRab18-A (denoted as GFP-Rab18-mutA) and another with silent mutations at the site targeted by shRab18-B (GFP-Rab18-mutB). As shown in Figure 3E, expression of GFP-Rab18-mutA rescued HCV replication in OR6 replicon cells transduced with shRab18-A, while GFP-Rab18-mutB did not. Rab18 protein knockdown and GFP-Rab18 expression were confirmed by immunoblotting (Figure 3F).

### Overexpression of Rab18 enhances HCV infectious particle production without affecting replication

**Figure 4:**
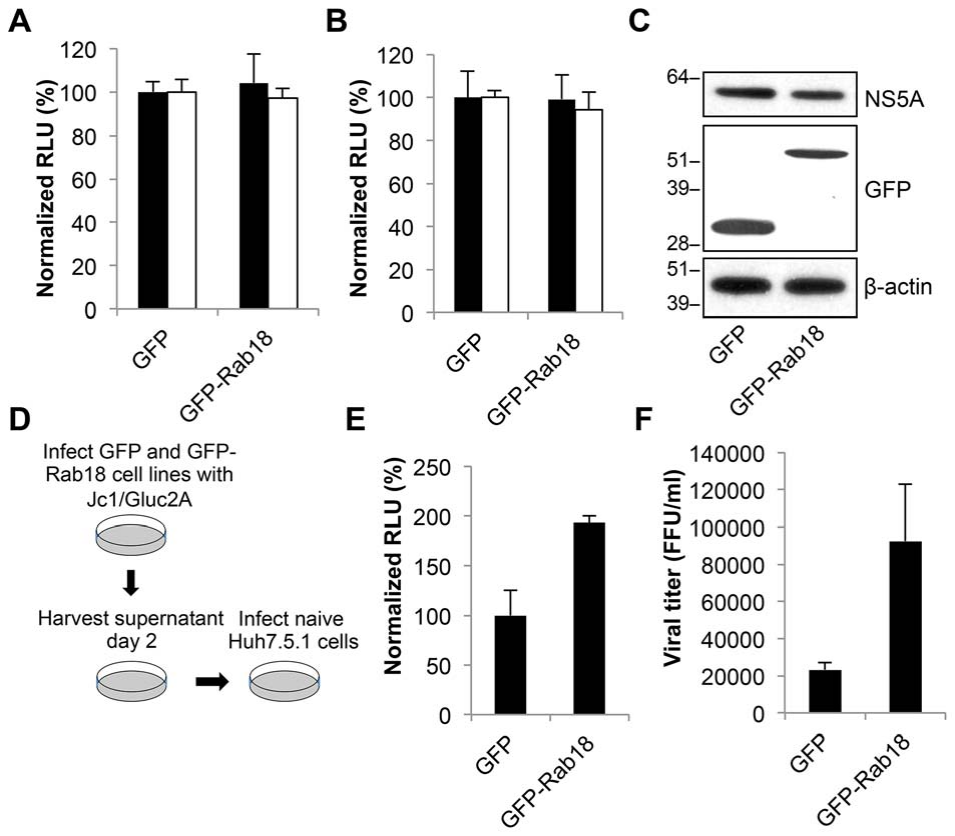
Rab18 overexpression enhances HCV infectious particle production. **A.** OR6 replicon cells were transduced with lentiviral vectors expressing either GFP or GFP-Rab18. 96 hr post-transduction, the cells were harvested for viral *Renilla* luciferase activity (black bars) and cell viability (white bars) measurement. All values are normalized to OR6 cells transduced with GFP alone and represent means ± SD of three independent experiments. **B.** Huh7.5.1 cells stably expressing GFP or GFP-Rab18 were infected with Jc1/Gluc2A at an MOI of 1. 96 hr post-infection, *Gaussia* luciferase activity (black bars) and cellular viability (white bars) were measured. All values are normalized to cells transduced with GFP and represent means ± SD of three independent experiments. **C.** Cell lysates from the previous experiment were subjected to immunoblotting for NS5A, GFP, and β-actin. **D and E.** GFP or GFP-Rab18 stable Huh7.5.1 cell lines were infected with Jc1/Gluc2A at an MOI of 1. Cell culture medium was harvested at day 4 post-infection, then used to infect naïve Huh7.5.1 cells. *Gaussia* luciferase signal was measured at 72 hr post-infection. All values are normalized to cells stably expressing GFP and represent means ± SD of three independent experiments. **F.** Stable cell lines expressing GFP or GFP-Rab18 were infected with JFH-1 at an MOI of 3. Five days post-infection, the secreted virus titer was determined using a focus-forming assay in naive Huh7.5.1 cells. All values represent means ± SD of three independent experiments.

The localization of Rab18 to lipid droplets and the importance of HCV-LD interactions in virion assembly suggested that Rab18 might play a role in infectious particle production. As Rab18 is known to promote the interaction of ER membranes with LDs, we hypothesized that Rab18 overexpression might enhance the production of infectious particles. Huh7.5.1 cells stably expressing GFP or GFP-Rab18 were infected with Jc1/Gluc2A, and replication was determined at 72 hr post-infection by measuring *Gaussia* luciferase activity in the culture supernatant. GFP-Rab18 overexpression did not alter OR6 replication (Figure 4A) or Jc1/Gluc2A replication (Figure 4B) compared to GFP overexpression. Although HCV replication is impaired by Rab18 silencing, these results suggest that Rab18 levels are not limiting for HCV replication in cell culture. Immunoblotting confirmed expression of both GFP and GFP-Rab18 at similar levels (Figure 4C). As depicted in Figure 4D, relative quantification of released infectious virus was determined by infection of naive Huh7.5.1 cells followed by measurement of *Gaussia* luciferase activity at 72 hr postinfection. GFP-Rab18 overexpression enhanced secretion of infectious Jc1/Gluc2A by nearly 2-fold compared to GFP overexpression (Figure 4E). We also observed significantly increased infectious particle release in Huh7.5.1 cells overexpressing GFP-Rab18 using the JFH-1 strain of HCV. (Figure 4F).

### Rab18 silencing reduces production of infectious HCV particles

**Figure 5:**
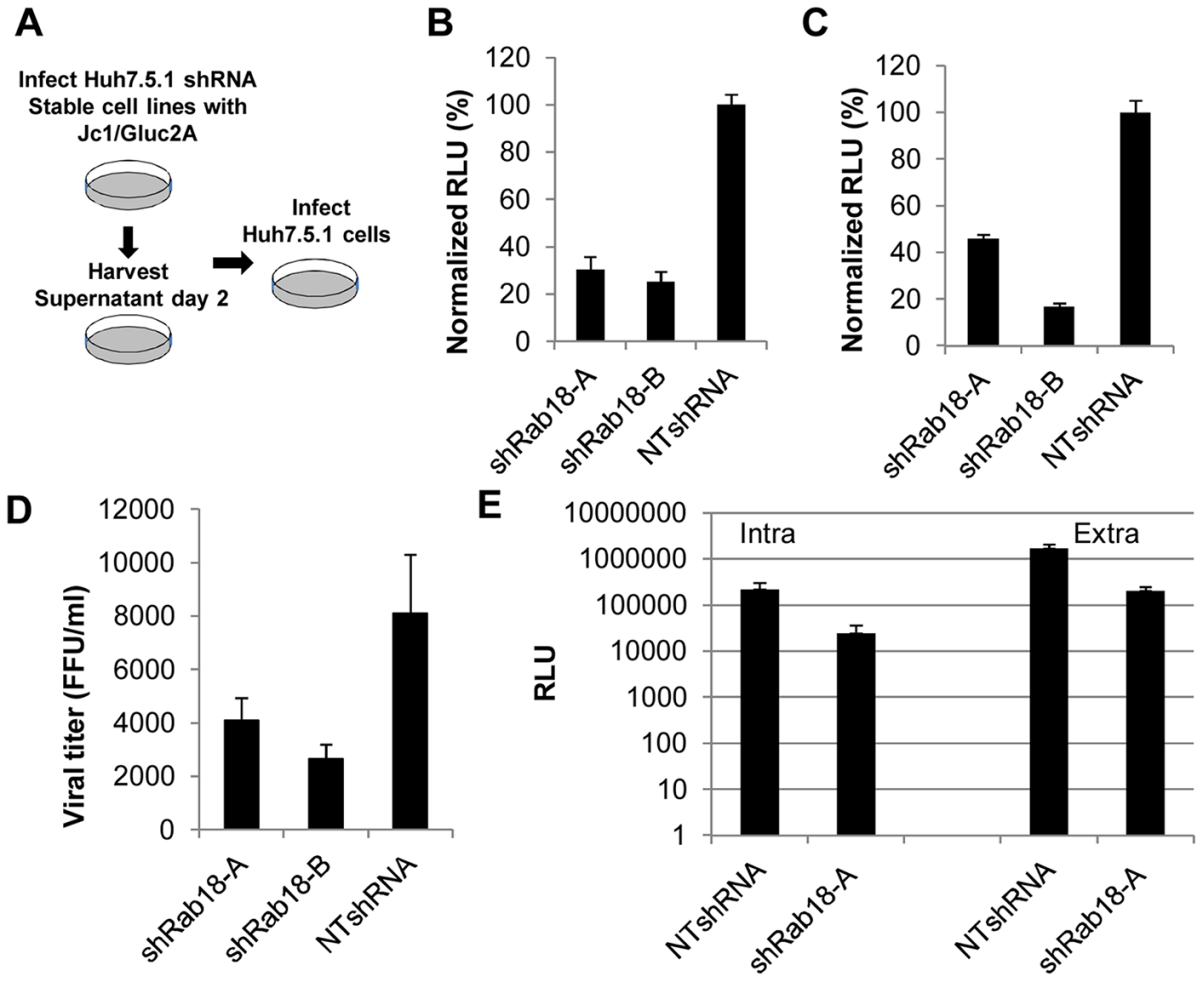
Rab18 silencing reduces the production of infectious HCV particles. **A.** Schematic of viral assembly/secretion assay. **B.** Huh 7.5.1 cell lines stably expressing shRab18-A, -B, or NTshRNA were infected with Jc1/Gluc2A at an MOI of 1. Cell culture medium was collected at day 2 postinfection, then used to infect naïve Huh7.5.1 cells. *Gaussia* luciferase activity was measured at 72 hr post-infection. These values were normalized to Huh7.5.1 cells infected with supernatant from NTshRNA-expressing cells. Values represent means ± SD of three independent experiments. **C.** Huh7.5.1 cells lines stably expressing shRab18-A, -B, or NTshRNA were transfected with *in vitro* transcribed Jc1/Gluc2A RNA. Relative quantitation of infectious particle release was performed as described above. **D.** Effect of Rab18 silencing on wild type JFH-1 secretion. Stable shRNA-expressing cell lines were infected with the JFH-1 strain of HCV at an MOI of 3. Five days post-infection, the secreted virus titer was determined using a focus-forming assay in naive Huh7.5.1 cells. Values represent means ± SD of three independent experiments. **E.** Huh 7.5.1 cell lines stably expressing shRab18-A, -B, or NTshRNA were infected with Jc1/Gluc2A at an MOI of 1. At 72 hr post-infection, the cell culture supernatant was collected for quantitation of released extracellular virus as described above. The infected cell monolayer was washed, trypsinized, and then the pellet was subjected to three rounds of freeze-thawing to release intracellular virus particles, which were then quantitated by infection of naïve Huh7.5.1 cells as described for extracellular infectious virus.

As shown in Figure 5A, cell pools stably expressing a nontargeting shRNA or Rab18-targeting shRNA were infected with Jc1/Gluc2A and cell culture medium containing released virus was harvested at 48 hr postinfection. Relative quantification of released infectious virus was determined by infection of naive Huh7.5.1 cells followed by measurement of *Gaussia* luciferase activity at 72 hr postinfection. Jc1/Gluc2A infectious particle release was reduced by Rab18 silencing by 69.5 and 74.9% percent in the shRab18-A and -B cell pools, respectively, compared to the nontargeting shRNA cell pool (Figure 5B). To exclude a possible effect of Rab18 silencing on HCV entry, we transfected *in vitro*-transcribed Jc1/Gluc2A RNA into Huh7.5.1 cells stably expressing a nontargeting shRNA or Rab18-targeting shRNAs, and observed similar reductions of HCV particle release by Rab18 knockdown (Figure 5C).

We also tested the effect of Rab18 silencing on infectious particle release using the JFH-1 strain of HCV. Absolute quantitation of released infectious virus by a focus-forming unit assay showed that infectious particle release was inhibited by Rab18 silencing by 50 and 67% percent in the shRab18-A and -B cell pools, respectively, compared to the nontargeting shRNA cell pool (Figure 5D).

We also quantitated the relative amounts of intracellular and extracellular infectious virus in Jc1/Gluc2A infected stable cell lines. Rab18 knockdown decreased levels of both intracellular and extracellular infectious virus to a similar extent (Figure 5E). Although we cannot definitively determine that Rab18 silencing blocks particle assembly because it also inhibits viral replication, the observation that Rab18 overexpression enhances infectious particle production without affecting replication does suggest that Rab18 may play a role in particle production.

Density gradient fractionation demonstrated that the buoyant densities of the fractions with peak extracellular and intracellular infectivity in HCV-infected cells were similar between shRab18 and NTshRNA expressing cell lines (Figure S2A), suggesting that Rab18 knockdown does not grossly alter the physical properties of infectious virions. The specific infectivity of the fractions with peak extracellular and intracellular infectivity was determined; the extracellular and intracellular specific infectivities measured from shRab18-expressing cells were not significantly different from NTshRNA-expressing cells (Figure S2B). These specific infectivities are consistent with previous studies [42].

As the inhibition of HCV production by Rab18 knockdown could simply be due to a block of LD biogenesis, we tested the effect of Rab18 silencing on LD accumulation induced by oleic acid loading. However, Rab18 silencing had no effect on mean LD diameter in Huh7.5.1 cells or on LD accumulation in oleic acid-loaded cells (Figure S3) compared to cells stably expressing a nontargeting shRNA. Therefore, Rab18 supports HCV infectious particle production by a mechanism other than simple regulation of LD volume.

### HCV NS5A binds to active forms of Rab18

**Figure 6:**
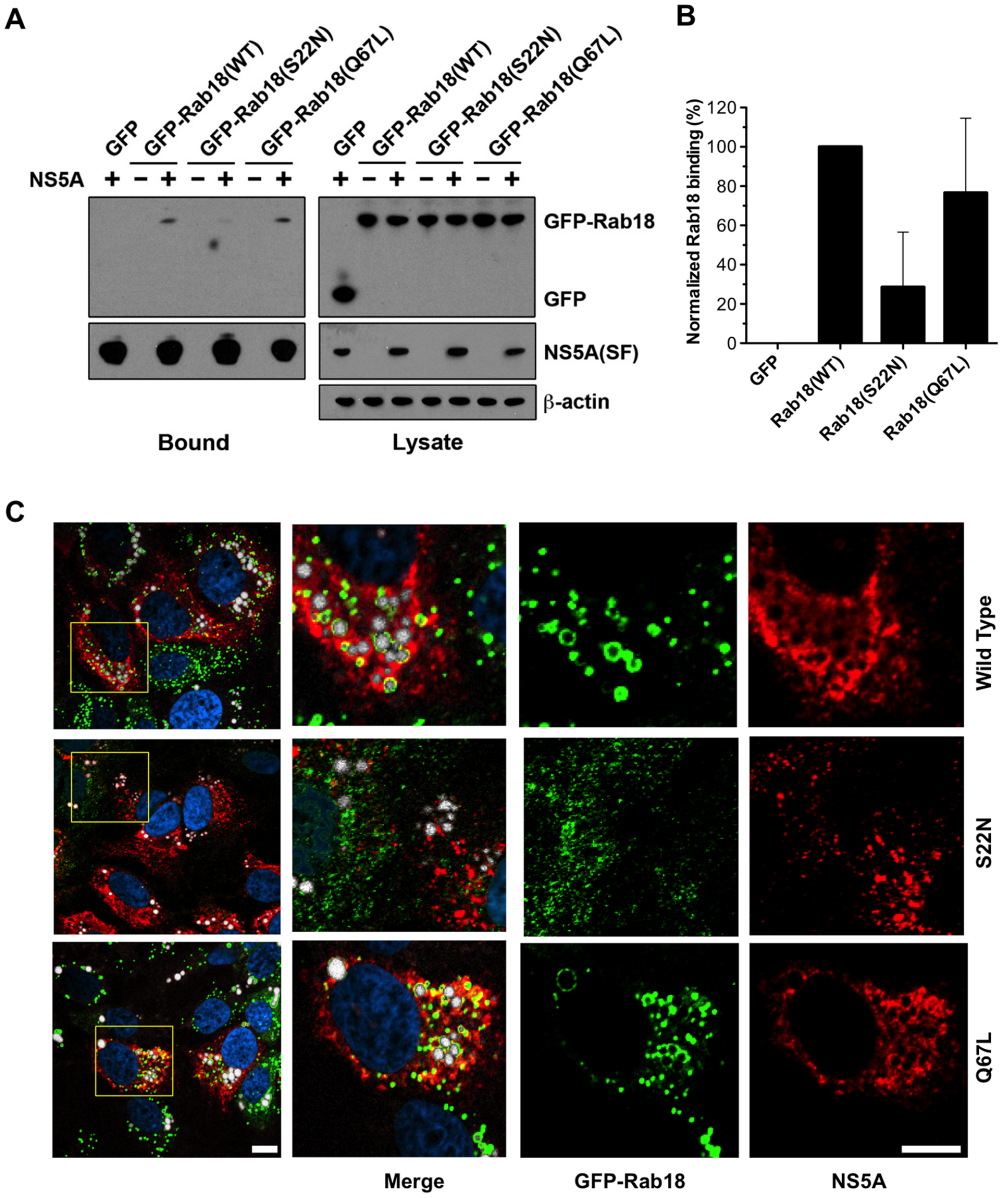
HCV NS5A binds to active forms of Rab18. (**A**) 293T cells were co-transfected with expression plasmids encoding NS5A(SF) with tandem Strep and FLAG tags (see Figure 1A) and either GFP, GFP-Rab18, GFP-Rab18(S22N), or GFP-Rab18 (Q67L). NS5A(SF) was pulled down from cell lysates with Streptactin-Sepharose at 48 hr post-transfection. Cell lysates (right panels) and eluted proteins (left panels) were subjected to Western blotting for NS5A, GFP, and β-actin. **B.** Quantitation of bound GFP-Rab18 was performed by quantitation of chemiluminescent signals on immunoblots. Results are normalized to wild-type GFP-Rab18 and represent means ± SD of four independent experiments. **C.** Distribution of NS5A and GFP-Rab18 (wild-type, S22N, or Q67L) in stable cell lines infected with JFH-1. Cells were immunostained for GFP (green) and NS5A (red) with counterstaining for lipid droplets (HCS LipidTox Deep Red, false-colored white) and DNA (DAPI, blue). Bar, 10 µm.

Rab proteins, like other small GTPases, cycle between an inactive, GDP-bound conformation and an active, GTP-bound conformation. We asked whether NS5A binds preferentially to a particular Rab18 conformation. 293T cells were cotransfected with expression plasmids encoding NS5A(SF) and either GFP, GFP-Rab18, the constitutively GDP-bound mutant GFP-Rab18(S22N), or the constitutively GTP-bound mutant GFP-Rab18(Q67L). These two mutants have been previously characterized [33]. NS5A(SF) and bound proteins were recovered by binding to Streptactin-Sepharose. Expression levels of the GFP-Rab18 constructs were similar as determined by immunoblotting of cell lysates (Figure 6A). As shown in Figure 6A, NS5A(SF) bound to GFP-Rab18 but not to GFP alone. In addition, the GDP-bound Rab18(S22N) mutant bound more weakly to NS5A(SF) than wild-type Rab18 or the GTP-bound Rab18(Q67L) mutant (Figures 6A and B). These results confirm that Rab18 interacts with NS5A in cells; furthermore, they suggest that NS5A has a higher affinity for the GTP-bound conformation of Rab18 than the GDP-bound conformation.

We examined the distribution of NS5A in HCV-infected cells overexpressing wild-type Rab18 or the S22N or Q67L mutants (Figure 6C). As expected, NS5A colocalized with wild-type Rab18 or the activated Rab18(Q67L) mutant around LDs. In contrast, overexpression of Rab18(S22N), which does not localize to LDs, was associated with reduced NS5A-LD association. Specifically, 94±4.5% (251 of 267 LDs) and 95±4.7% (177 of 186) of LDs in GFP-Rab18(wt) and GFP-Rab18 (Q67L) expressing cells were NS5A positive, respectively, while 46±5.4% (65 of 140) of LDs in GFP-Rab18(S22N) overexpressing cells were NS5A positive. Quantitation of lipid droplets in oleic acid-loaded Huh7.5.1 stably expressing GFP or GFP-Rab18 (wild type, S22N, or Q67L) demonstrated no significant alterations in the average LD diameter (Figure S4). As the number of cells overexpressing GFP-Rab18(S22N) in stable cell lines was consistently low despite multiple attempts, we were unable to study the effect of GFP-Rab18(S22N) overexpression on HCV particle release (data not shown). Overexpression of the constitutively active GFP-Rab18(Q67L) mutant did result in enhanced HCV particle release to a degree similar to that seen with wild-type GFP-Rab18 overexpression (data not shown).

### Modulation of Rab18 levels affects association of NS5A, NS3, and HCV RNA, but not core protein, with lipid droplets

**Figure 7:**
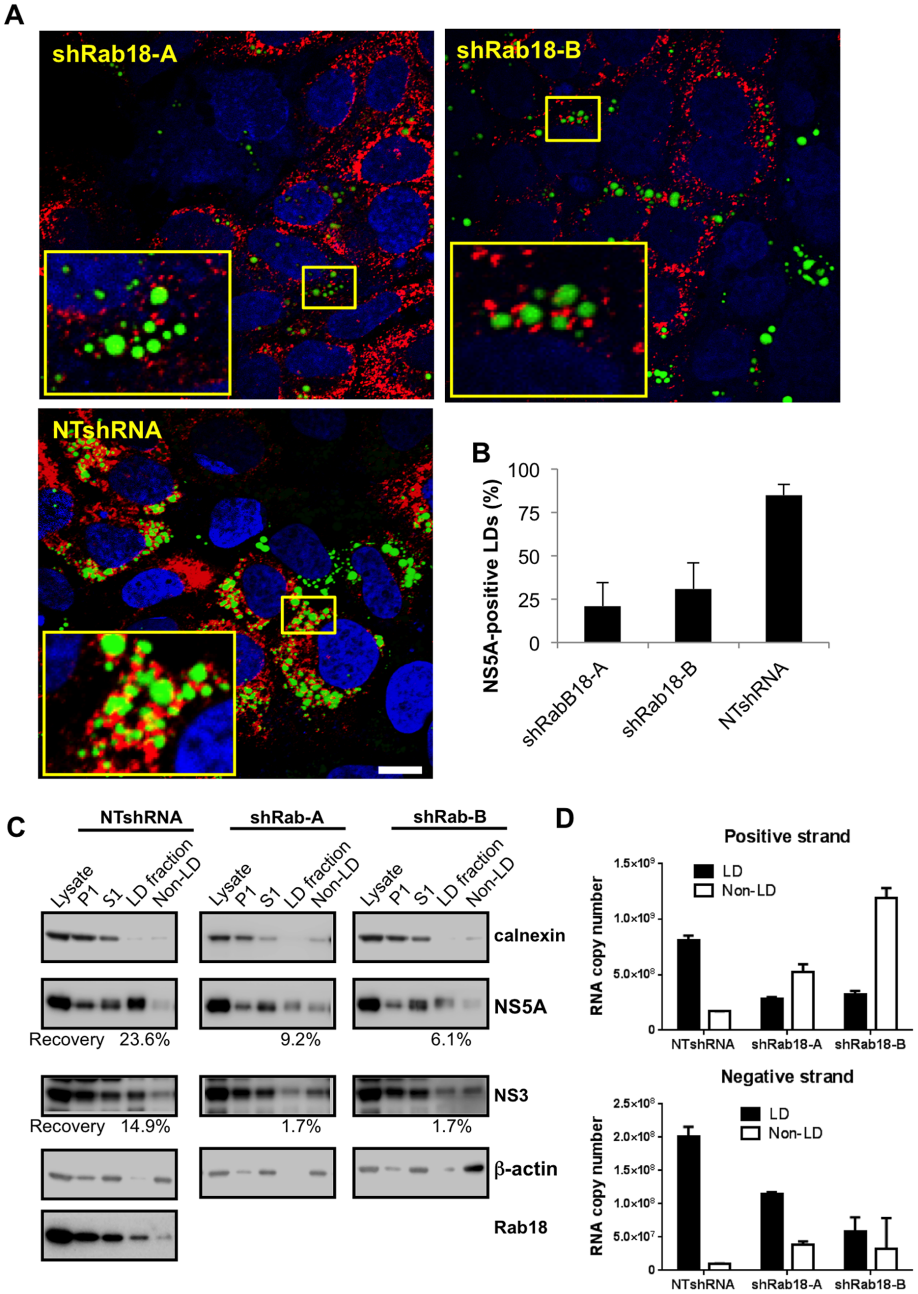
Rab18 modulates the association of multiple HCV components with lipid droplets. **A.** Huh7.5.1 cells stably expressing a nontargeting shRNA or two independent Rab18 shRNAs were infected with JFH-1 and then processed for immunofluorescence staining of NS5A (red). LDs were visualized with BODIPY 493/503 (green) and nuclei were counterstained with DAPI (blue). Bar, 10 µm. **B.** Quantitation of the percentage of LDs (visualized by BODIPY 493/503 staining) positive for NS5A in cells expressing Rab18 shRNAs or a nontargeting shRNA. All values represent means ± SD of two independent experiments in which a minimum of 1000 LDs were counted from each cell line. **C.** Association of NS5A and NS3 with LDs in Rab18-silenced cells. Stable cell lines expressing a nontargeting shRNA (left panels) or shRNAs targeting Rab18 (middle and right panels) were infected with JFH-1 at an MOI of 3. Five days later, cells were homogenized and a postnuclear supernatant was centrifuged at 16,000× g for 15 min, resulting in a P1 pellet and an S1 supernatant. The S1 supernatant was separated by sucrose density gradient centrifugation. LDs and associated proteins float to the top of the gradient, while cytosol and other denser cellular components remain at the bottom of the gradient. Samples from the P1, S1, top and bottom fractions were subjected to immunoblotting for NS5A, NS3, calnexin as an ER marker, β-actin as a cytoskeletal/cytosolic marker, and Rab18. Chemiluminescent immunoblot detection was used to quantitate NS5A and NS3 band intensities; the recovery of NS5A and NS3 in the LD-associated top fractions is expressed as a percentage of the total input NS5A and NS3 in the S1 supernatant. This is a representative immunoblot from four independent experiments. **D.** Total positive- and negative-strand HCV RNA in the LD and non-LD fractions was quantitated by strand-specific quantitative RT-PCR. Samples were diluted to approximately 10^6^ copies of input RNA to maximize specificity. Values are means ± SD of triplicate measurements.

Given the known ability of Rab18 to approximate ER membranes to lipid droplets, we hypothesized that Rab18 might also help physically tether NS5A-positive membranes to LDs. We tested this hypothesis by first infecting Huh7.5.1 cell lines stably expressing Rab18 shRNAs or a nontargeting shRNA with JFH-1. Silencing of Rab18 was associated with a decrease in NS5A association with LDs compared to cells expressing a nontargeting shRNA (Figure 7A). We quantitatively assessed the decrease in NS5A-LD association by Rab18 silencing in cells infected with HCV, and found that Rab18 silencing led to a significant decrease in the proportion of LDs that stained positive for NS5A. In nontargeting shRNA-expressing cells, 85±6% of LDs stained positive for NS5A, while in two independent Rab18 shRNA-expressing cell lines, only 21±15% and 26±13% of LDs were NS5A-positive (Figure 7B).

As NS5A is a component of HCV replication platforms, we asked whether the association of other HCV components with LDs could also be modulated by Rab18. Huh7.5.1 cells stably expressing a nontargeting shRNA or a Rab18-targeting shRNA were infected with the JFH-1 strain of HCV, homogenized, and the post-nuclear supernatant was subjected to low-speed centrifugation. The supernatant (S1) was then fractionated by density gradient centrifugation to isolate LDs. Similar amounts of HCV RNA from nontargeting or Rab18 shRNA-expressing cells were loaded onto the density gradients (Figure S5A). Fractions from the top of the density gradient (enriched in LDs) and from the bottom of the density gradient (enriched in cytosol and other intracellular membranes) were immunoblotted for NS5A, NS3, calnexin, actin, and Rab18 (Figure 7C). Most of the ER membranes (as shown by immunoblotting for the ER marker calnexin), NS5A, and NS3 were removed by the 16,000× *g* centrifugation step. As expected, Rab18 was enriched in the LD fraction from NTshRNA-expressing cells compared to the non-LD fraction.

In control cells, nearly a quarter of the NS5A in the S1 supernatant could be recovered in the LD-enriched top gradient fraction, with a smaller amount of NS3 found in the top fraction. On the other hand, we observed substantially lower recoveries of LD-associated NS5A and NS3 in cells silenced for Rab18. We then quantitated the amount of LD-associated HCV RNA using strand-specific quantitative RT-PCR, and found that cells silenced for Rab18 had decreases in both LD-associated negative-strand and positive-strand RNA compared to cells expressing nontargeting shRNA, with some increase in the amount of negative and positive-strand RNA isolated from non-LD fractions (Figure 7D). These findings indicate that Rab18 promotes not only the association of NS5A, but also other components of the HCV replication complex, with LDs.

The association of HCV core protein with LDs is believed to be essential for particle assembly [29], [43]. We examined the distribution of core protein in JFH-1 infected cells stably expressing nontargeting or Rab18-targeting shRNAs. The association of core with LDs was not altered by Rab18 silencing or overexpression of Rab18 (wt or mutant) (Figures S6 and S7), indicating that core protein association with LDs is not regulated by Rab18.

## Discussion

In this study, a strategy combining affinity purification with mass spectrometry to identify novel host factors that associate with NS5A in cells infected with a cell-culture infectious HCV strain revealed that Rab18, a lipid droplet-specific Rab GTPase, interacts with NS5A. A similar strategy using Strep-tag affinity purification of NS5A has been previously used to identify oxysterol-binding protein (OSBP) as an NS5A-interacting protein [44]. That study used a subgenomic replicon, as opposed to our use of a fully infectious HCV construct, which may have enhanced our ability to detect host proteins involved in virion production. In addition, we used SILAC to reduce the identification of false-positive protein interactions.

Other Rab GTPases have been described to promote HCV replication. The endocytic Rab5 and Rab7 proteins appear to facilitate HCV genome replication [45]–[47], perhaps by inducing autophagy [48]. A proteomic analysis of detergent-resistant membrane (DRM) fractions, which are believed to be enriched in HCV replication complexes, isolated from Huh7 cell lines expressing a full-length HCV replicon cells found that HCV-replicating cells had increased levels of Rab7 associated with DRMs compared to control Huh7 cells [49]. Of note, this study also found that Rab18 was enriched on DRM fractions from replicon-expressing cells, although its role in the HCV life cycle was not directly tested. In addition to Rab5 and Rab7, other work has suggested a role for Rab1 and TBC1D20, a Rab1 GTPase-activating protein, in HCV replication. TBC1D20 binds to NS5A [50], and depletion of TBC1D20 or Rab1 by RNA interference reduces HCV RNA levels [50], [51]. In the absence of NS5A, neither TBC1D20 nor Rab1 is found on lipid droplets; it appears that NS5A recruits both of these proteins to LDs [25].

In contrast, both endogenous and overexpressed Rab18 localize to LDs in the absence of HCV [32], [33]. In HCV-infected cells, both endogenous and overexpressed Rab18 colocalize with NS5A around LDs. Rab18 silencing and expression of dominant-negative Rab18 reduce association of NS5A with LDs. NS3 and HCV RNA (both negative and positive-strand) cofractionation with LDs is also reduced by Rab18 silencing. Furthermore, immunoelectron microscopy identifies NS5A-positive membrane profiles that are in close association with Rab18-positive LDs in Rab18-overexpressing cells. Taken together, these findings suggest that Rab18 not only recruits NS5A but also membranous sites of HCV genome replication (also known as “membranous webs”) to lipid droplets. This is consistent with two previous reports that Rab18 overexpression leads to the close apposition of ER membranes to LDs [33], [52]. In these studies, electron microscopy of Rab18-overexpressing cells demonstrated wrapping of thin membrane cisternae around LDs, some of which were continuous with the rough ER or themselves studded with ribosomes. Ozeki et al. proposed that LD-associated ER membranes represented a specialized ER region that they termed the lipid droplet-associated membrane, or LAM; this may be analogous to other ER domains that appose other intracellular membranes such as the plasma membrane and mitochondria. In contrast to the effect of Rab18 modulation on the association of NS5A and other membranous web components with LDs, we did not observe any effect of Rab18 modulation on the localization of HCV core protein to LDs, suggesting that the interaction between core protein and LDs is Rab18-independent.

The current model proposing that Rab18 recruits ER membranes to LDs may be relevant to the HCV life cycle. While it is generally accepted that HCV replication occurs on ER-derived membranes, it is unclear where progeny HCV genomes are packaged into virions. While LDs appear to play a critical role in the process of HCV assembly, it remains uncertain whether HCV assembly occurs at the LD membrane itself or with closely-associated ER membranes (reviewed in [53]). Assembled HCV virions then traffic through the secretory pathway with glycosylation of the envelope proteins, indicating that HCV virions must then enter the ER lumen during or following assembly. Attempts at identifying the site of HCV virion assembly by microscopic techniques have so far been unsuccessful, likely because the rate of assembly events is likely to be low and because HCV viral particles are heterogeneous in appearance [54]. Nevertheless, an important unanswered question is how HCV RNA replication is physically coupled to virion production and subsequent entry into the cellular secretory pathway.

Our observations support a model in which Rab18 helps promote interaction between LDs and HCV membranous webs through a direct association between NS5A and the active, GTP-bound form of Rab18. A somewhat unexpected finding was that Rab18 silencing inhibited HCV replication without inhibiting LD biogenesis in response to fatty acid loading. While LDs have been shown to be important for HCV particle assembly, there has been relatively little evidence that LDs support HCV genome replication. Indirect support for this possibility comes from studies demonstrating that Rab1 supports HCV replication and that a dominant negative Rab1 mutant suppresses LD formation. In addition, during preparation of this manuscript it was reported that knockdown of the LD-associated protein TIP47 inhibits HCV replication in addition to infectious particle release [55]. Further studies are needed to determine the mechanisms by which cellular LDs assist in HCV replication.

We speculate that the Rab18-promoted interaction between HCV membranous webs and LDs may also enhance virion assembly by bringing sites of replication in close physical proximity to sites of virion assembly, either on the LD surface or on membranes apposed to the LD. Because Rab18 silencing also affects HCV genome replication, the decrease in intracellular and extracellular virus titers seen in Rab18 silenced cells cannot be definitively ascribed to inhibition of particle assembly. However, the observation that Rab18 overexpression increases infectious virus production without altering replication does support a possible role for Rab18 in virus production. Note that this model does not exclude the possibility of additional protein-protein interactions that might mediate LD-membranous web interactions. For example, the HCV core protein also appears to play a key role in recruiting HCV nonstructural proteins to LDs [26]. On the other hand, we and others have found that HCV NS5A localizes to LDs when expressed alone in the absence of core protein [24], [25], [27] and (Salloum and Tai, unpublished observations), indicating that core protein is not necessary for NS5A-LD interaction. Intriguingly, a small-molecule inhibitor of NS5A relocalizes NS5A from the ER to LDs in cells expressing a genotype 1b replicon [27], suggesting that the interaction between NS5A and LDs may also be regulated by NS5A conformation. It has been proposed that phosphorylation of NS5A may direct a switch from viral replication to virion assembly [56]; future studies will examine the potential role of Rab18 interaction with NS5A in modulating this transition.

## Materials and Methods

### Cell lines and cell culture

Huh7.5.1 cells [57], a subline of Huh7 human hepatoma cells that is highly permissive for HCV replication, and 293T cells were grown in Dulbecco's modified Eagle's medium (DMEM) supplemented with 10% fetal bovine serum (FBS), nonessential amino acids, 100 U/mL of penicillin, and 100 µg/mL of streptomycin. OR6 cells containing a full-length genotype 1b HCV replicon with a *Renilla* luciferase reporter gene have been described elsewhere [39].

### Antibodies

Antibodies used included those directed against calnexin (mouse monoclonal; Sigma-Aldrich, St Louis, MO), FLAG epitope tag (mouse monoclonal clone M2; Sigma-Aldrich), GFP (rabbit monoclonal, Cell Signaling Technology, Beverly, MA), HCV core (mouse monoclonal clone 6G7; Dr. Harry Greenberg, Stanford, CA), HCV NS5A (mouse monoclonal clone 9E10; Dr. Charles Rice, Rockefeller University, New York, NY), Rab18 (rabbit polyclonal; Proteintech, Chicago, IL), and β-Actin (mouse monoclonal; Sigma-Aldrich). HCS LipidTOX Deep Red neutral lipid stain, BODIPY 493/503, DAPI, and Alexa Fluor conjugated secondary antibodies for microscopy experiments were purchased from Invitrogen (Carlsbad, CA, USA).

### DNA constructs

HCV clones used in this work include the cell culture infectious genotype 2a clone JFH-1 [58] and Jc1/Gluc2A [36], which encodes a *Gaussia* luciferase reporter gene in the J6/JFH-1 chimeric genome known as Jc1 [35]. Jc1(SF) refers to Jc1 with insertion of a tandem affinity purification tag (Strep-tag and FLAG) into NS5A described elsewhere [17].

Two independent Rab18 shRNA lentiviral vectors for Rab18 knockdown were obtained from the TRC shRNA library [59] (TRCN0000021981 and TRCN0000021983, Sigma-Aldrich). A nontargeting shRNA in the same pLKO.1 backbone (Addgene plasmid 1864) was used as a negative control.

GFP-Rab18 lentiviral expression constructs were generated from plasmids encoding EGFP-human Rab18 and the mutants S22N and Q67L [33] by PCR amplification of the GFP-Rab18 coding sequences using the primer pair 5′-CGCGTGCCGCCACCATGGTGAGCAAG-3′, and 5′-CGTACGTTATAACACAGAGCAATA ACCACCACAGG-3′. The amplicons were subcloned using MluI and BsiWI restriction sites into the pSMPUW-IRES-blasticidin lentiviral expression vector (Cell Biolabs, San Diego, CA). We modified this vector to include the MluI and BsiWI restriction sites in the multiple cloning site; details of vector construction are available upon request. EGFP was subcloned into the same vector as a negative control. All constructs were confirmed by sequencing. The GFP-Rab18-mutA construct was generated by introducing three silent mutations into the TRCN0000021981 shRNA target region using mutagenic primers 5′-CAGGCCTTCATTACGGTCTACTTCACGATTTTCCTTATCG -3′, and 5′-CGTGAAGTAGAC CGTAATGAAGGCCTGAAATTTGC -3′, while the GFP-Rab18-mutB construct contained silent mutations in the TRCN0000021983 shRNA target region introduced using mutagenic primers 5′-GACATCATAAACAAGAATTACACCCTGTGCACCTCTATAATAG -3′ and 5′- GGTGCACAGGGTGTAATTCTTGTTTATGATGTCACAAGAAG -3′.

Lentiviral expression constructs encoding full-length NS5A(SF), NS5A(SF) lacking the N-terminal amphipathic helix, and NS5A(SF) lacking domains I, II, or III were generated by PCR amplification from the JFH-1 NS5A sequence and subcloning into our modified pSMPUW-IRES-blasticidin vector using MluI and EcoRI restriction sites. The domain III-deleted construct had a C-terminal SF tag immediately following domain II. All constructs were confirmed by sequencing.

### Stable Isotope Labeling with Amino Acids in Cell Culture (SILAC) and mass spectrometry

Huh 7.5.1 cells were cultured in SILAC medium (DMEM with high glucose, sodium pyruvate, without L-arginine or L-lysine, Cambridge Isotope Laboratories, Andover, MA) with 10% dialyzed FBS, 1% penicillin-streptomycin, and either “light” L-arg and L-lys (LAA) or “heavy” L-arg-^13^C_6_, ^15^N_4_ and L-lys-^13^C_6_,^15^N_2_ (HAA) at concentrations of 146 mg/L and 84 mg/L, respectively. Unlabeled L-proline (200 mg/L) was also added to inhibit arginine-to-proline conversion [60]. All amino acids were obtained from Sigma-Aldrich. Cells were grown for 6 days (two passages) in “heavy” or “light” medium for complete metabolic labeling of the cells grown in “heavy” medium prior to viral infection. Pilot experiments confirmed 97% incorporation of heavy lysine and arginine into the proteome of Huh7.5.1 cells grown under these conditions with negligible arginine to proline conversion (data not shown). Cells were then plated at a density of 10^6^ cells per 10 cm dish 24 hr before infecting LAA-treated cells with Jc1 and HAA-treated cells with Jc1(SF) virus at a MOI of 1. 2 days post-infection, Jc1 and Jc1(SF)-infected cells were split 1∶5 into medium containing “light” or “heavy” medium, respectively. 4×10^7^ cells were harvested from each condition at day 6 postinfection and lysed in 50 mM Tris pH 7.5, 150 mM NaCl, 1 mM EDTA, and 0.5% Triton X-100 with Halt protease inhibitor cocktail (Pierce, Rockford, IL). After centrifugation at 21,000× g for 15 min at 4°C to remove cell nuclei and insoluble material, 1.3 mg of “light” and “heavy” labeled protein isolated from Jc1 and Jc1(SF) infected cells, respectively, were mixed together and then incubated with Streptactin-Sepharose (IBA, Göttingen, Germany) for 1 hr at 4°C. Unbound material was removed by 3 washes in lysis buffer with 0.5% Triton X-100 followed by 3 washes in lysis buffer with 0.1% Triton X-100. Bound proteins were eluted with lysis buffer with 0.1% Triton X-100 and 4 mM biotin at 4°C. To remove Triton X-100 and concentrate the eluted protein, proteins were precipitated by deoxycholic acid/trichloroacetic acid followed by acetone washes and then were solubilized in SDS-PAGE loading buffer. Proteins were separated on SDS-PAGE and visualized by Coomassie Blue staining.

The stained gel was excised into ten equally sized segments. In-gel digestion was performed by washing with 25 mM ammonium bicarbonate followed by acetonitrile, reducing with 10 mM dithiothreitol at 60°C followed by alkylating with 50 mM iodoacetamide at RT. Samples were digested with trypsin at 37°C for 4 hr followed by formic acid quenching and direct analysis without further processing. Each gel digest was analyzed by nano LC-MS/MS with a Waters nanoACQUITY UPLC system (Waters, Milford, MA) interfaced to a LTQ Orbitrap Velos (Thermo Fisher Scientific, Waltham, MA). Peptides were loaded on a trapping column and eluted over a 75 µm analytical column at 350 nL/min; both columns were packed with Jupiter Proteo resin (Phenomenex, Torrance, CA). The mass spectrometer was operated in data-dependent mode, with MS performed in the Orbitrap at 60,000 FWHM resolution and MS/MS performed in the LTQ. The fifteen most abundant ions were selected for MS/MS. MaxQuant software v1.0.13.13 was used to recalibrate MS data, filter database search results at the 1% protein and peptide false discovery rate, and calculate SILAC heavy∶light ratios. Protein and peptide identifications were performed using Mascot (Matrix Science, Boston, MA), which was configured to include Carbamidomethyl (C) as a fixed modification and the following variable modifications: Oxidation (M), Acetyl (N-term), Pyro-Glu (N-term Q), Deamidation (N,Q), (^13^C_6_,^15^N_2_) K and (^13^C_6_,^15^N_4_) R. Data were searched with a peptide mass tolerance of 10 ppm and fragment mass tolerance of 0.5 Da. The database used was a combination of Uniprot human and virus databases that were reversed, concatenated and appended with common contaminants.

### Production of lentiviral vectors and stable cell lines

VSV-G pseudotyped lentiviral vectors (for shRNA or protein expression) were produced by cotransfection of 293T cells with the packaging vectors (Addgene plasmid 12260) and pMD2.G (Addgene plasmid 12259). Lentiviral supernatants were harvested at 48 and 74 hr post-transfection, 0.45 µm filtered, and stored at −80°C. Target cells were transduced with lentiviral particles for 4 hr in the presence of 8 µg/mL polybrene (Sigma).

For generation of knockdown Rab18 cell lines, Huh7.5.1 cells were transduced with lentivirus encoding two independent shRNAs targeting Rab18 or a nontargeting shRNA. Stable knockdown pools were isolated by puromycin selection. For stable expression of GFP-Rab18, Huh7.5.1 cells were transduced with lentiviral expression vectors encoding GFP or GFP-Rab18 (wild-type, S22N, or Q67L) followed by blasticidin selection.

### Immunofluorescence microscopy

Cells grown on poly-D-lysine coated glass coverslips were rinsed in PBS and fixed in 4% paraformaldehyde in PBS for 15 min at RT followed by quenching in 50 mM NH4Cl in PBS for 10 min at RT. Blocking and antibody incubations were performed in 10% fetal bovine serum and 0.1% saponin in PBS for 1 hr at RT. Blocking was followed by incubation with primary antibody in blocking buffer for 1 hr in RT followed by 4 washes in PBS for 5 min each. Secondary antibody detection was performed with Alexa Fluor 488- and 568- conjugated anti-rabbit and anti-mouse antibodies (Invitrogen). Lipid droplets were visualized using either LipidTOX Deep Red or BODIPY 493/503 (Invitrogen) according to the manufacturer's directions. After 4 washes in PBS for 5 minutes each, coverslips were mounted with Prolong Gold with DAPI (Invitrogen) and viewed on an Olympus FluoView FV500 or Nikon A1 laser scanning confocal microscope with sequential scanning mode to limit crosstalk between fluorochromes. For the purposes of quantitation, we defined NS5A association with lipid droplets as a minimum of 25% of the LD diameter associated with NS5A.

### Immunoelectron microscopy

Control uninfected Huh7.5.1 cells stably expressing GFP-Rab18 or cells infected with HCV for 5 days were fixed in 8% paraformaldehyde in phosphate buffer and processed for frozen sectioning according to published protocols [32]. Although glutaraldehyde fixation resulted in superior ultrastructural preservation, immunoreactivity of glutaraldehyde-fixed specimens with two different NS5A antibodies was inadequate (data not shown). Ultrathin frozen sections were labeled with rabbit antibodies to GFP [61] and the mouse 9E10 antibody to NS5A, followed by goat anti-mouse antibodies conjugated to 10 nm gold particles, and goat anti-rabbit antibodies conjugated to 15 nm gold particles (BBI, Cardiff, UK). Sections were viewed in a JEM-1011 microscope (Jeol, Japan).

### Oleic acid loading

Selected cell lines were incubated with 180 µM oleic acid-BSA (Sigma) for 24 hr. Cells were fixed and processed for LD staining with BODIPY 493/503 (for shRNA-expressing cells) or HCS LipidTox Deep Red (for GFP-expressing cells) with DAPI nuclear counterstaining as described above. Quantitation of LD size and number was performed using NIH ImageJ version 1.47 g software. Briefly, images were background-subtracted and thresholded by Otsu's method. LDs in close proximity to one another were separated by watershed segmentation, and their Feret diameters were quantitated using the ImageJ particle analysis function.

### HCV replication and infectious virus production assays

For measurement of HCV replication, *Renilla* luciferase activity in OR6 replicon cells was measured using the *Renilla* Luciferase Assay System (Promega, Madison, WI) and a Synergy 2 microplate reader equipped with reagent injectors (Biotek, Winooski, VT) using the manufacturer's directions. Cell viability was determined by measurement of cellular ATP content using the CellTiter-Glo Assay (Promega).

Cells were infected with JFH-1 or Jc1/Gluc2A at an MOI of 3 for 4 hr at 37°C. Alternatively, Jc1/Gluc2A RNA was transcribed *in vitro* as described in [62] and the *in vitro* transcribed RNA was transfected into cells with TransiT mRNA reagent (Mirus Bio, Madison, WI) as described in [63]. The cell monolayers were washed with complete medium to remove input virus, and the cell culture medium was harvested at 48 hr postinfection. Secreted JFH-1 virus in cell culture supernatants was quantitated by a focus-forming unit (FFU) assay. Each sample was serially diluted tenfold and used to infect naive Huh7.5.1 cells in 96-well plates. 3 days postinfection, cells were fixed with methanol and stained for HCV core; the titer was calculated by counting infected cell foci and expressed in FFU/mL.

For cells infected with Jc1/Gluc2A, cell culture medium collected at 48 hr post-infection was used to infect naïve Huh7.5.1 cells. Gaussia luciferase activity was measured at 72 hr post-infection as described in [36].

For determination of intracellular versus extracellular infectious virus titer, selected cell lines were infected with Jc1/Gluc2A virus at an MOI of 1. At 72 hr postinfection, supernatants containing extracellular virus were harvested. Infected cells were washed to remove residual extracellular virus, collected by trypsinization and centrifugation, and the cell pellet was resuspended in a volume of medium equal to the extracellular virus supernatant. After three rounds of freeze-thawing to release intracellular virus particles followed by centrifugation to remove cell debris, the released intracellular virus and the extracellular virus supernatants were used to infect naïve Huh7.5.1 cells. Gaussia luciferase activity was measured at 72 hr post-infection.

Density gradient fractionation of HCV virions was performed as previously described by [42] with the following minor modifications: 1 mL samples were layered on top of a discontinuous gradient of 6%-12%-18%-24%-30%-40% iodixanol and centrifuged at 30,000 rpm (111,000× g_avg_) for 18 hr at 4°C in an SW41 rotor.

### Rab18-NS5A binding assay

293T cells were cotransfected with GFP-Rab18 (or GFP as a negative control) and Strep/FLAG-tagged NS5A constructs using Fugene HD (Promega) according to the manufacturer's directions. 48 hr after transfection, cells were washed with phosphate-buffered saline (PBS) and lysed in 50 mM Tris pH 7.5, 150 mM NaCl, 1 mM EDTA, and 0.5% Triton X-100 with protease inhibitors. After centrifugation at 21,000× g for 15 min at 4°C to remove insoluble material, equal amounts of protein from clarified lysates were incubated with Streptactin-Sepharose (IBA, Gottingen, Germany) for 1 hr at 4°C to recover Strep/FLAG-tagged NS5A. Unbound material was removed by washing in lysis buffer followed by washing in lysis buffer with 0.1% Triton X-100. Bound proteins were eluted with 4 mM biotin in lysis buffer with 0.1% Triton X-100 at 4°C and then separated on SDS-PAGE for immunoblotting. Quantitation of chemiluminescence signals from immunoblots was performed on an Odyssey Fc imager (Li-Cor, Lincoln, NE).

### Cofractionation of lipid droplets with NS5A and HCV RNA

Isolation of lipid droplet-enriched fractions from cells was performed by density gradient centrifugation. Briefly, selected cell lines were infected with JFH-1 and harvested at day 5 post-infection. The cell pellet was resuspended in homogenization buffer (10 mM Tris pH 8.0, 135 mM NaCl, 10 mM KCl, 5 mM MgCl2) supplemented with protease inhibitors (Pierce). The cell suspension was homogenized with 10 strokes of a ball-bearing homogenizer (Isobiotec, Heidelberg, Germany; 10 µm clearance) and then centrifuged at 1,000× *g* for 10 min at 4°C to remove nuclei and unbroken cells. The postnuclear supernatant was then spun at 16,000× *g* for 15 min at 4°C. The pellet (P1) was saved for subsequent immunoblotting. The supernatant (S1) was mixed with an equal volume of 1.04 M sucrose in homogenization buffer and transferred to a 5 mL ultracentrifuge tube (Beckman Coulter, Indianapolis, IN). This was overlaid with 1 mL of homogenization buffer and the discontinuous gradient was centrifuged at 45,000 rpm for 1.5 hr at 4°C in a MLS-50 rotor (Beckman Coulter)

After the centrifugation, the LD fraction was recovered from the top of the gradient. The collected LD fraction, the bottom fraction, and the S1 supernatant were used for immunoblotting analysis. Quantitation of chemiluminescence signals from immunoblots was performed on an Odyssey Fc imager (Li-Cor, Lincoln, NE).

LD-associated HCV RNA was isolated using RNeasy Mini columns (QIAGEN) and strand-specific quantitative RT-PCR was performed as previously described [64] with the following minor modifications. The Tag-RC1 and RC1 primers were changed to the sequences GGCCGTCATGGTGGCGAATAA G**C**CTAGCCATGGCGTTAGTA and G**C**CTAGCCATGGCGTTAGTA, respectively, to match the JFH-1 5′NTR sequence. Quantitation of the cDNA was performed using the DyNAmo HS SYBR Green qPCR kit (Finnzyme, Espoo, Finland) and primers described in [41].

## Supporting Information

Figure S1 (**A**) Localization of endogenous Rab18 and NS5A in OR6 cells stably expressing a full-length genotype 1b HCV replicon. OR6 cells were fixed and stained for endogenous Rab18 (left panel) and NS5A (middle panel). Nuclei were counterstained with DAPI (blue). Bar, 10 µm. (**B**) Association of core protein with LDs in JFH-1 and Jc1(SF)-infected cells. Huh7.5.1 cells infected with JFH-1 (upper panels) or Jc1(SF) (lower panels) were fixed and stained for core protein (red, left panels) and lipid droplets (green, middle panels). Nuclei were counterstained with DAPI (blue). Bar, 10 µm.
(TIF)

Figure S2 (**A**) Effect of Rab18 silencing on buoyant densities of extracellular and intracellular virions. Stable cell lines expressing NTshRNA or shRab18-A were infected with Jc1/Gluc2A for 5 days prior to collection of cell culture supernatant and intracellular virions by freeze-thawing. Separation of virions was performed on iodixanol density gradients, and the infectivity of the collected fractions was determined by infection of naïve Huh7.5.1 cells followed by *Gaussia* luciferase measurement. Values are expressed as means ± SD from two independent experiments. (**B**) Extracellular and intracellular specific infectivity of the peak infectivity fractions isolated from stable cells lines expressing NTshRNA or shRab18-A.
(TIF)

Figure S3 Effect of Rab18 silencing on lipid droplets. (**A**) Stable cell lines expressing NTshRNA, shRab18-A, or shRab18-B were treated with BSA alone (left panels) or loaded with 180 µM of oleic acid-BSA complexes (right panels) for 24 hr and then fixed and processed for LD staining using BODIPY 493/503 with DAPI nuclear counterstaining. Bar, 10 µm. (**B**) Lipid droplet Feret diameters in cells without oleic acid loading were calculated using NIH ImageJ software; over 6000 lipid droplets in randomly selected microscope fields were quantitated per condition. Values are expressed as means ± SD.
(TIF)

Figure S4 Effect of GFP-Rab18 overpression (wt and mutants) on lipid droplet biogenesis. (**A**) Stable cell lines expressing GFP or GFP-Rab18 (wt, S22N, or Q67L) were treated with BSA alone (left panels) or loaded with 180 µM of oleic acid-BSA complexes (right panels) for 24 hr and then fixed and processed for LD staining using HCS LipidTox Deep Red (false-colored purple) with DAPI nuclear counterstaining. Bar, 10 µm. (**B**) Lipid droplet Feret diameters in cells without oleic acid loading were calculated using NIH ImageJ software; over 2500 lipid droplets in randomly selected microscope fields were quantitated per condition. Values are expressed as means ± SD.
(TIF)

Figure S5 (**A**) Strand-specific HCV RNA quantitation to confirm similar amounts of HCV RNA in the S1 supernatants used for density gradient fractionation in Figure 7. Stable cell lines expressing a nontargeting shRNA (left panels) or shRNAs targeting Rab18 (middle and right panels) were infected with JFH-1 at an MOI of 3. Five days later, cells were homogenized and a postnuclear supernatant was centrifuged at 16,000× g for 15 min, resulting in a P1 pellet and an S1 supernatant. The S1 supernatant was diluted to approximately 10^6^ input strands for strand-specific RNA quantitation in order to maximize assay specificity. (**B**) Strand specificity of the positive and negative-strand HCV RNA quantitation assay. The left-sided plots show the results of the positive-strand quantitation assay using the indicated mass of positive-strand synthetic RNA generated by *in vitro* transcription (upper left) and negative-strand synthetic RNA (lower left). The right-sided plots show the results of the negative-strand quantitation assay using the indicated mass of negative-strand synthetic RNA (upper right) and positive-strand synthetic RNA (lower right).
(TIF)

Figure S6 Effect of Rab18 silencing on core association with LDs. (**A**) Stable cell lines expressing NTshRNA, shRab18-A, or shRab18-B were infected with JFH-1 and then immunostained for HCV core protein (red). Counterstaining was performed for LDs (BODIPY 493/503, green) and DNA (DAPI, blue). Bar, 10 µm. (**B**) The percentage of LDs with associated core immunostaining in HCV-infected cells is plotted as means ± SD. A total of 161, 104, and 87 LDs were scored from NTshRNA, shRab18-A, and shRab18-B stable cell lines, respectively.
(TIF)

Figure S7 Effect of GFP-Rab18 overpression (wt and mutants) on core distribution. Stable cell lines expressing GFP or GFP-Rab18 (wt, S22N, or Q67L) were infected with JFH-1 and then immunostained for HCV core protein (red) or GFP (green). Counterstaining was performed for DNA (DAPI, green). Note that endogenous Rab18 is not visualized in these images. Bar, 10 µm.
(TIF)
