# Supplementary figures and images for: Rab18 Binds to Hepatitis C Virus NS5A and Promotes Interaction between Sites of Viral Replication and Lipid Droplets
Source: PLoS Pathog. 2013 Aug 1;9(8):e1003513. doi: 10.1371/journal.ppat.1003513 (PMC3731246; doi:10.1371/journal.ppat.1003513)

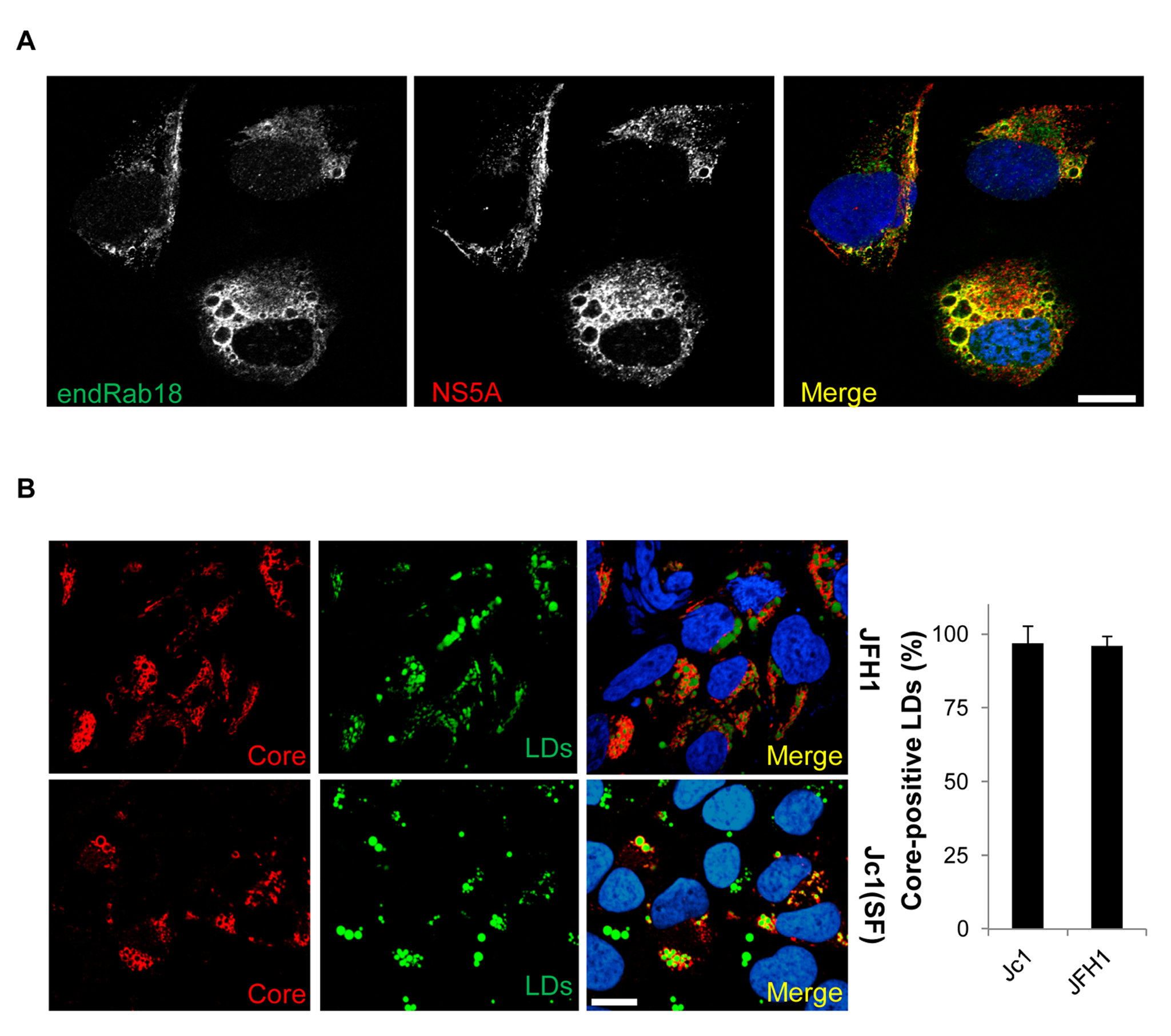

Supplement: Figure S1 — (A) Localization of endogenous Rab18 and NS5A in OR6 cells stably expressing a full-length genotype 1b HCV replicon. OR6 cells were fixed and stained for endogenous Rab18 (left panel) and NS5A (middle panel). Nuclei were counterstained with DAPI (blue). Bar, 10 µm. (B) Association of core protein with LDs in JFH-1 and Jc1(SF)-infected cells. Huh7.5.1 cells infected with JFH-1 (upper panels) or Jc1(SF) (lower panels) were fixed and stained for core protein (red, left panels) and lipid droplets (green, middle panels). Nuclei were counterstained with DAPI (blue). Bar, 10 µm. (TIF) [file ppat.1003513.s001.tif]

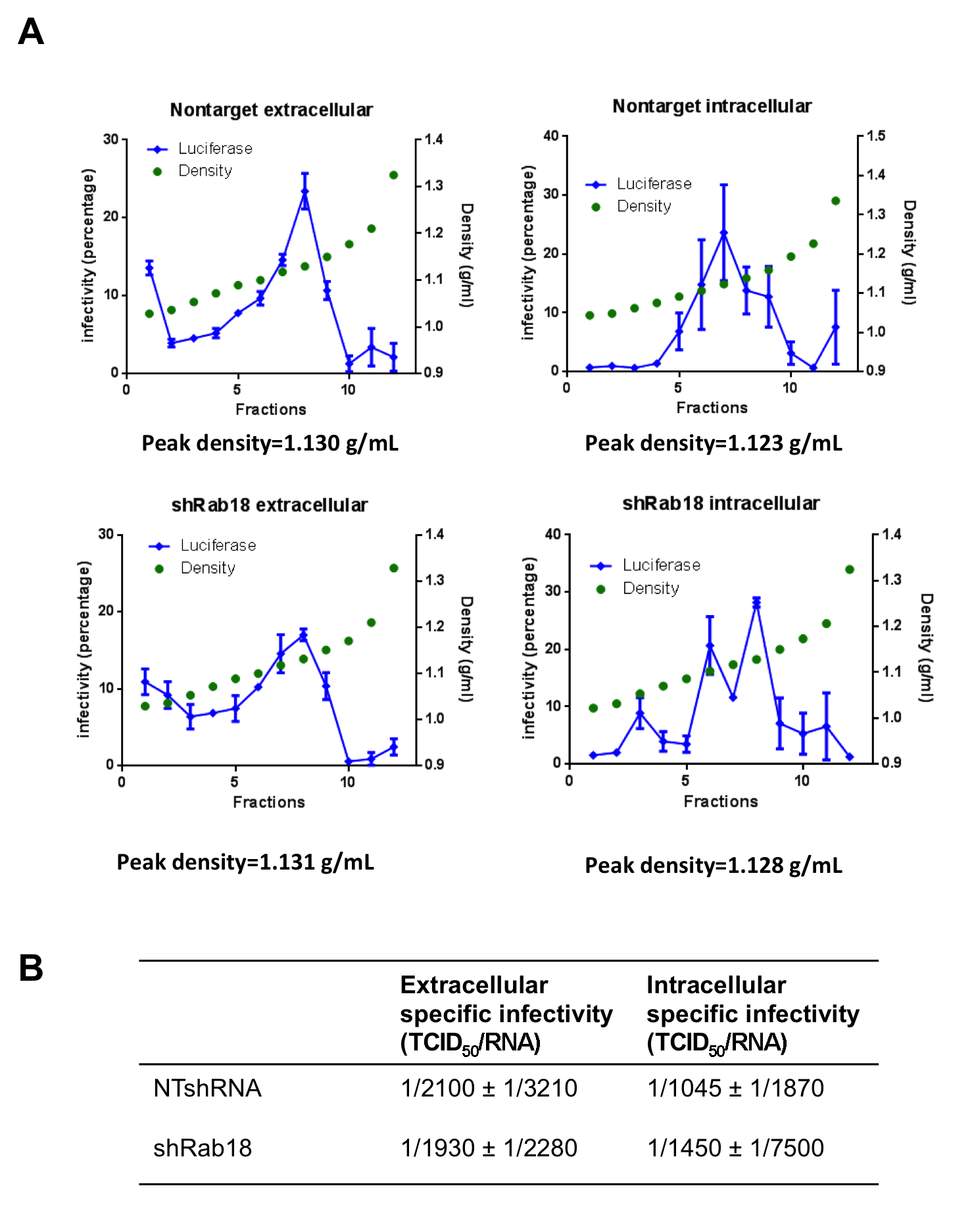

Supplement: Figure S2 — (A) Effect of Rab18 silencing on buoyant densities of extracellular and intracellular virions. Stable cell lines expressing NTshRNA or shRab18-A were infected with Jc1/Gluc2A for 5 days prior to collection of cell culture supernatant and intracellular virions by freeze-thawing. Separation of virions was performed on iodixanol density gradients, and the infectivity of the collected fractions was determined by infection of naïve Huh7.5.1 cells followed by Gaussia luciferase measurement. Values are expressed as means ± SD from two independent experiments. (B) Extracellular and intracellular specific infectivity of the peak infectivity fractions isolated from stable cells lines expressing NTshRNA or shRab18-A. (TIF) [file ppat.1003513.s002.tif]

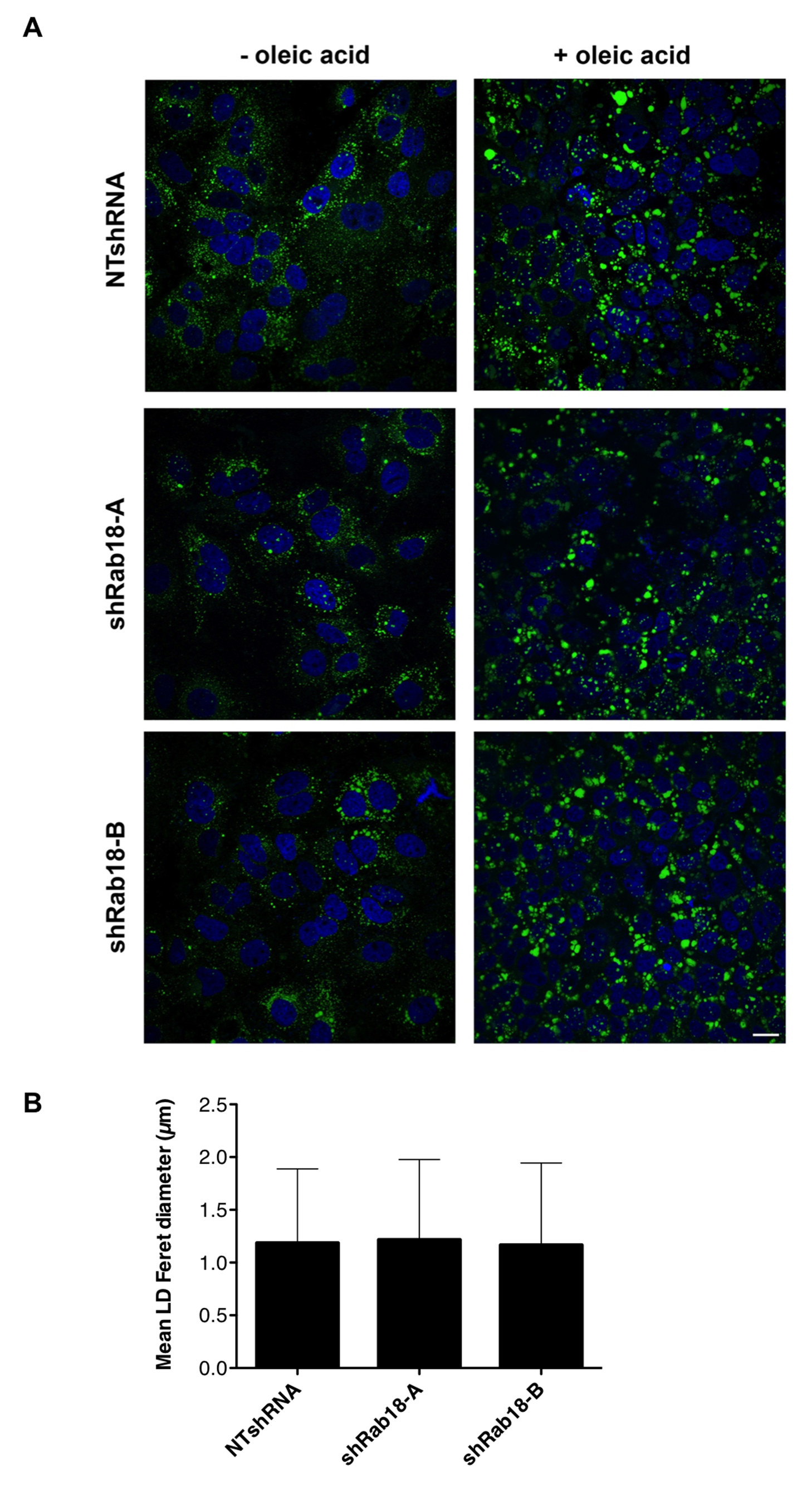

Supplement: Figure S3 — Effect of Rab18 silencing on lipid droplets. (A) Stable cell lines expressing NTshRNA, shRab18-A, or shRab18-B were treated with BSA alone (left panels) or loaded with 180 µM of oleic acid-BSA complexes (right panels) for 24 hr and then fixed and processed for LD staining using BODIPY 493/503 with DAPI nuclear counterstaining. Bar, 10 µm. (B) Lipid droplet Feret diameters in cells without oleic acid loading were calculated using NIH ImageJ software; over 6000 lipid droplets in randomly selected microscope fields were quantitated per condition. Values are expressed as means ± SD. (TIF) [file ppat.1003513.s003.tif]

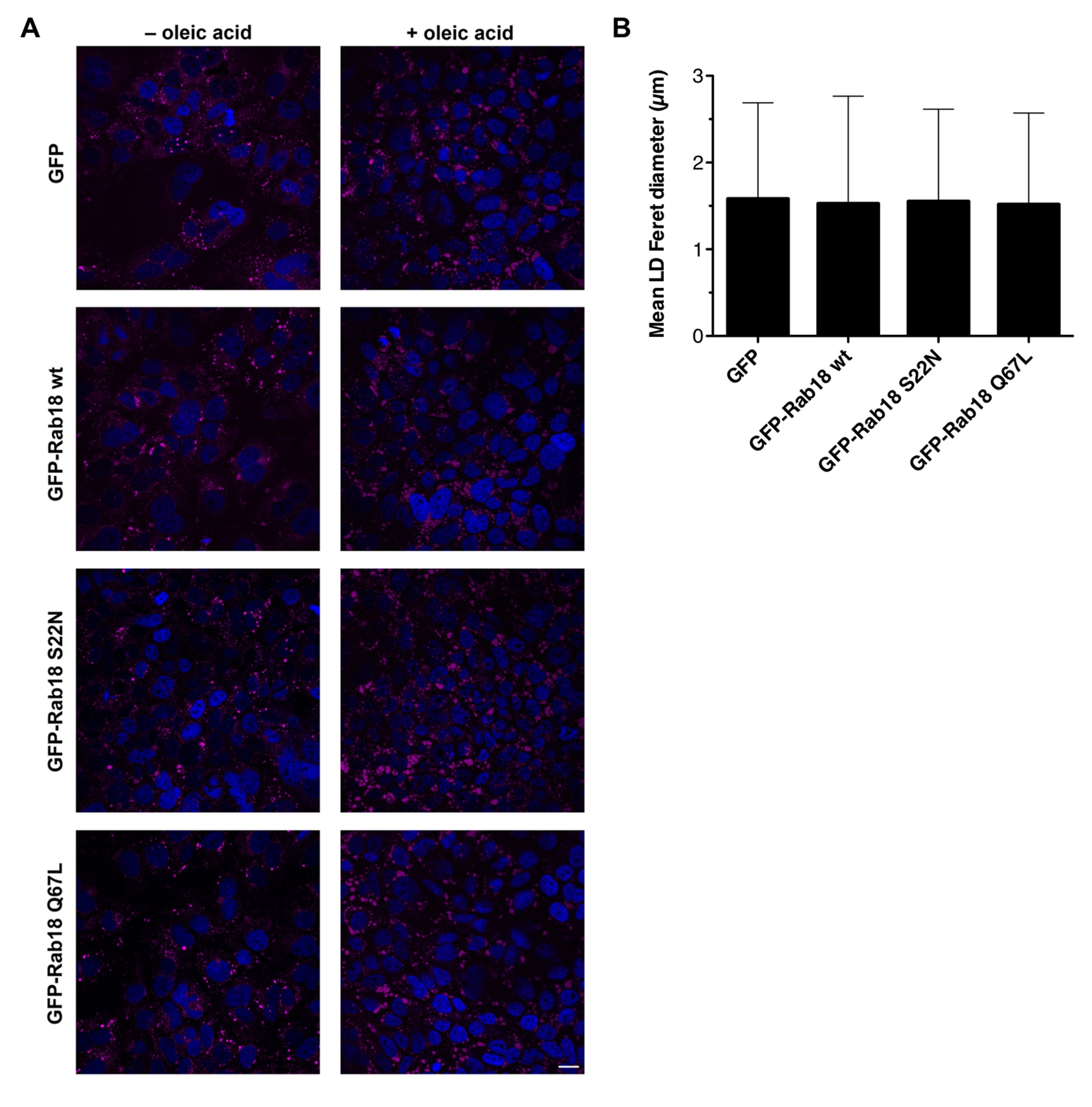

Supplement: Figure S4 — Effect of GFP-Rab18 overpression (wt and mutants) on lipid droplet biogenesis. (A) Stable cell lines expressing GFP or GFP-Rab18 (wt, S22N, or Q67L) were treated with BSA alone (left panels) or loaded with 180 µM of oleic acid-BSA complexes (right panels) for 24 hr and then fixed and processed for LD staining using HCS LipidTox Deep Red (false-colored purple) with DAPI nuclear counterstaining. Bar, 10 µm. (B) Lipid droplet Feret diameters in cells without oleic acid loading were calculated using NIH ImageJ software; over 2500 lipid droplets in randomly selected microscope fields were quantitated per condition. Values are expressed as means ± SD. (TIF) [file ppat.1003513.s004.tif]

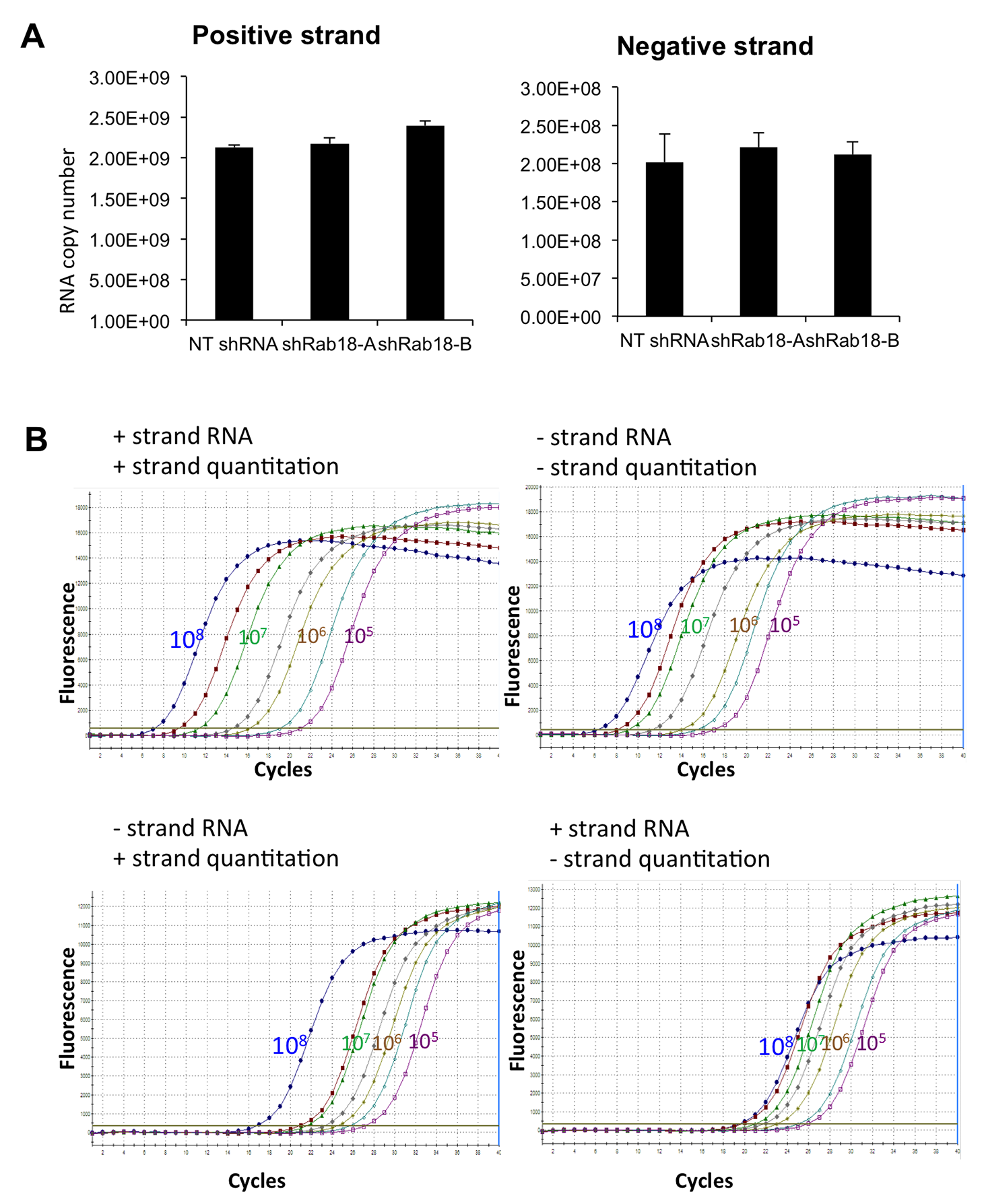

Supplement: Figure S5 — (A) Strand-specific HCV RNA quantitation to confirm similar amounts of HCV RNA in the S1 supernatants used for density gradient fractionation in Figure 7. Stable cell lines expressing a nontargeting shRNA (left panels) or shRNAs targeting Rab18 (middle and right panels) were infected with JFH-1 at an MOI of 3. Five days later, cells were homogenized and a postnuclear supernatant was centrifuged at 16,000× g for 15 min, resulting in a P1 pellet and an S1 supernatant. The S1 supernatant was diluted to approximately 106 input strands for strand-specific RNA quantitation in order to maximize assay specificity. (B) Strand specificity of the positive and negative-strand HCV RNA quantitation assay. The left-sided plots show the results of the positive-strand quantitation assay using the indicated mass of positive-strand synthetic RNA generated by in vitro transcription (upper left) and negative-strand synthetic RNA (lower left). The right-sided plots show the results of the negative-strand quantitation assay using the indicated mass of negative-strand synthetic RNA (upper right) and positive-strand synthetic RNA (lower right). (TIF) [file ppat.1003513.s005.tif]

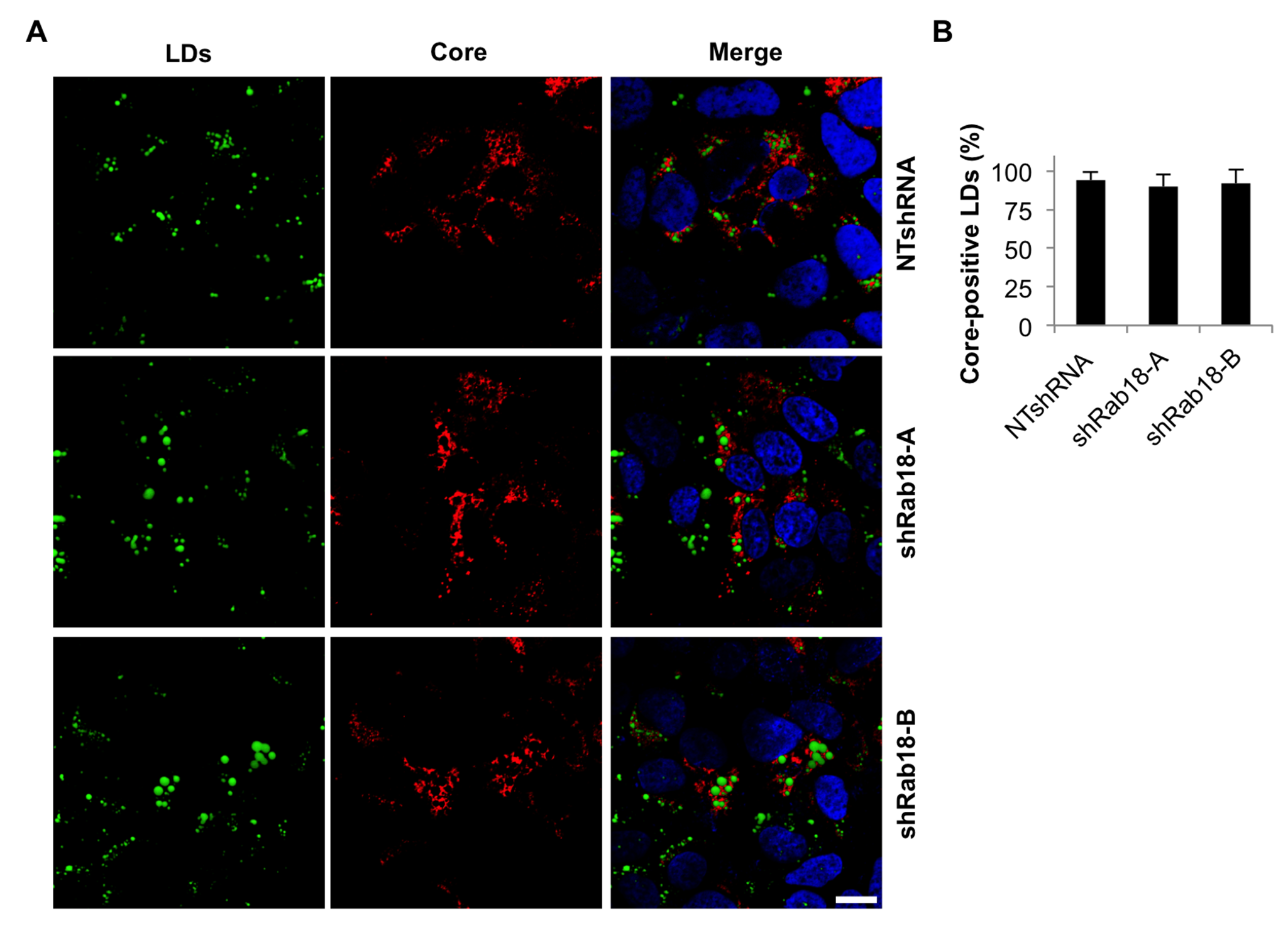

Supplement: Figure S6 — Effect of Rab18 silencing on core association with LDs. (A) Stable cell lines expressing NTshRNA, shRab18-A, or shRab18-B were infected with JFH-1 and then immunostained for HCV core protein (red). Counterstaining was performed for LDs (BODIPY 493/503, green) and DNA (DAPI, blue). Bar, 10 µm. (B) The percentage of LDs with associated core immunostaining in HCV-infected cells is plotted as means ± SD. A total of 161, 104, and 87 LDs were scored from NTshRNA, shRab18-A, and shRab18-B stable cell lines, respectively. (TIF) [file ppat.1003513.s006.tif]

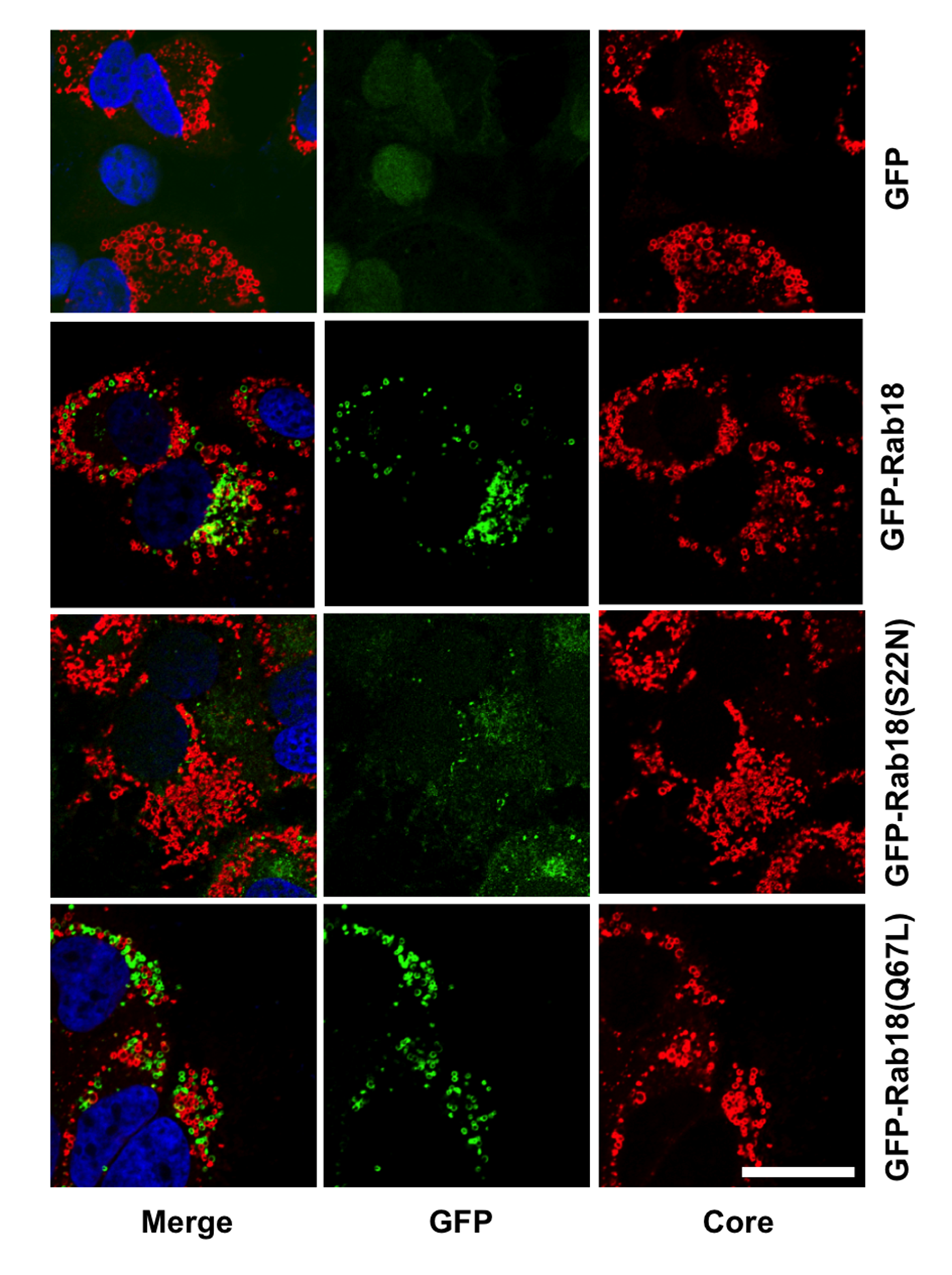

Supplement: Figure S7 — Effect of GFP-Rab18 overpression (wt and mutants) on core distribution. Stable cell lines expressing GFP or GFP-Rab18 (wt, S22N, or Q67L) were infected with JFH-1 and then immunostained for HCV core protein (red) or GFP (green). Counterstaining was performed for DNA (DAPI, green). Note that endogenous Rab18 is not visualized in these images. Bar, 10 µm. (TIF) [file ppat.1003513.s007.tif]
